# Complex patterns of divergence among green-sensitive (RH2a) African cichlid opsins revealed by Clade model analyses

**DOI:** 10.1186/1471-2148-12-206

**Published:** 2012-10-18

**Authors:** Cameron J Weadick, Belinda SW Chang

**Affiliations:** 1 Current Affiliation: Department of Evolutionary Biology, Max Planck Institute for Developmental Biology, Spemmanstr. 37, Tuebingen, 72076, Germany; 2 Department of Ecology and Evolutionary Biology, University of Toronto, 25 Harbord Ave, Toronto, Ontario, M5S 3G5, Canada; 3 Department of Ecology and Evolutionary Biology, Department of Cell and Systems Biology, and Centre for the Analysis of Genome Evolution and Function, University of Toronto, 25 Harbord Ave, Toronto, Ontario, M5S 3G5, Canada

**Keywords:** Codon substitution model, Visual pigment evolution, Nonsynonymous-to-synonymous substitution rate ratio, dN/dS, Clade model, Maximum likelihood, Gene family evolution

## Abstract

**Background:**

Gene duplications play an important role in the evolution of functional protein diversity. Some models of duplicate gene evolution predict complex forms of paralog divergence; orthologous proteins may diverge as well, further complicating patterns of divergence among and within gene families. Consequently, studying the link between protein sequence evolution and duplication requires the use of flexible substitution models that can accommodate multiple shifts in selection across a phylogeny. Here, we employed a variety of codon substitution models, primarily Clade models, to explore how selective constraint evolved following the duplication of a green-sensitive (RH2a) visual pigment protein (opsin) in African cichlids. Past studies have linked opsin divergence to ecological and sexual divergence within the African cichlid adaptive radiation. Furthermore, biochemical and regulatory differences between the RH2aα and RH2aβ paralogs have been documented. It thus seems likely that selection varies in complex ways throughout this gene family.

**Results:**

Clade model analysis of African cichlid RH2a opsins revealed a large increase in the nonsynonymous-to-synonymous substitution rate ratio (ω) following the duplication, as well as an even larger increase, one consistent with positive selection, for Lake Tanganyikan cichlid RH2aβ opsins. Analysis using the popular Branch-site models, by contrast, revealed no such alteration of constraint. Several amino acid sites known to influence spectral and non-spectral aspects of opsin biochemistry were found to be evolving divergently, suggesting that orthologous RH2a opsins may vary in terms of spectral sensitivity and response kinetics. Divergence appears to be occurring despite intronic gene conversion among the tandemly-arranged duplicates.

**Conclusions:**

Our findings indicate that variation in selective constraint is associated with both gene duplication and divergence among orthologs in African cichlid RH2a opsins. At least some of this variation may reflect an adaptive response to differences in light environment. Interestingly, these patterns only became apparent through the use of Clade models, not through the use of the more widely employed Branch-site models; we suggest that this difference stems from the increased flexibility associated with Clade models. Our results thus bear both on studies of cichlid visual system evolution and on studies of gene family evolution in general.

## Background

Gene duplication is known to play major roles in genomic and phenotypic evolution, and often precipitates divergent evolution of protein structure and function [1,2]. A number of models have been proposed to explain the retention and evolution of duplicated genes in the face of deleterious, pseudogenizing mutations [3-5]; these models differ in the predictions they make about post-duplication sequence evolution and, consequently, in how amenable they are to investigation. The classic neofunctionalization model [3], for example, predicts a fairly simple pattern of post-duplication protein sequence evolution, with one paralog diverging while the other retains the ancestral function under a regime of purifying selection. However, other models, such as the duplication-degeneration-complementation model [6] and the escape from adaptive conflict model [7,8], predict more complex forms of divergence. Furthermore, it is becoming increasingly recognized that divergent protein evolution can occur without duplication—that is, among orthologs—and that this can contribute to adaptive phenotypic evolution [9-11], contrary to classical assumptions [12]. Distinguishing among models of gene duplication, and determining the relative roles played by adaptive and non-adaptive processes in protein evolution, thus requires approaches that can accommodate complex patterns of sequence evolution.

Recent advances in codon substitution models that account for variation in site-specific selective constraint among multiple clades or lineages [13,14] provide a promising approach for distinguishing among models of gene duplication and evolution. Branch-site models [15,16] are commonly used to detect the signature of strong site-specific positive selection along a pre-specified lineage after gene duplication (cf. 'conserved-but-different' or 'Type II' divergence patterns) [17,18]. Clade models [19,20], meanwhile, can be used to detect more subtle differences in site-specific selective constraint among entire clades or partitions of a phylogeny (c.f. 'covarion-like' or 'Type I' divergence patterns) [17,18]. Clade models are not restricted to detecting strict cases of positive selection and can be used to consider variation among multiple clades simultaneously [21]. As such, Clade models may be better suited to detecting complex forms of divergence in selective constraint across gene families than the Branch-site models [22]. However, compared to the popular Branch-site models, Clade models have been relatively under used.

Species derived from recent adaptive radiations are intriguing systems for studying patterns of evolution at the molecular level, as rapid phenotypic evolution implies a comparable degree of change in the underlying genome [23,24]. The endemic and diverse cichlid fishes of the Rift Valley of eastern Africa are thought to be the result of multiple, young, adaptive radiations [25,26]. The high numbers of species found within the Rift Valley’s lakes and rivers, as well as the impressive degree of phenotypic variation present among closely related species, make these fishes ideal for studies on functional diversification and speciation. Recently, progress has been made associating adaptive phenotypic evolution in African cichlids with variation at the molecular level, for example with regard to jaw morphology or colour patterning, [27-30]. Perhaps most notably, a number of studies have linked ecological and sexual divergence among African cichlids to divergence in colour vision genes, the opsins [31,32]. Opsins form the protein component of visual pigments, the photosensitive compounds expressed in the rod and cone photoreceptor cells of the retina that absorb and transduce photons of light into the biochemical signals that ultimately underlie the visual sense [33-35]. Amino acid substitutions that affect the opsin’s retinal chromophore binding pocket can alter the pigment's absorbance spectrum, generating variation in spectral sensitivity [36,37]. Recent studies of African cichlid opsins have linked variation in opsin protein sequences and expression patterns to ecologically- and sexually-selected divergence among closely related populations and species [38,39] and, as a result, African cichlids have emerged as model systems for study of the molecular biology, evolution, and ecology of opsins [31,32].

Compared to most vertebrates, African cichlids possess a large number of opsin genes, with seven cone opsins and one rod opsin [40]. The resulting visual pigments vary in spectral sensitivity, with the wavelength of maximal absorbance (λ_max_) ranging from the ultraviolet (UV) to the yellow. Most of these opsins are evolutionarily ancient, with orthologs present in most teleosts, if not most vertebrates. However, the green-sensitive RH2aα and RH2aβ opsins are relatively young [41,42], and descend from a duplication event specific to the African cichlid clade [43]. Spectrophotometric study of RH2aα and RH2aβ pigments from *Oreochromis niloticus* (Nile tilapia) and *Metriaclima zebra* (Lake Malawi zebra mbuna), expressed *in vitro*, revealed functional divergence between the paralogs, with the RH2aα pigments red-shifted (λ_max_ ≈ 528 nm) compared to the RH2aβ pigments (λ_max_ ≈ 518 nm) [41,42]. However, it has proven difficult to broadly survey for variation in RH2aα/β λ_max_ via microspectrophotometry (MSP) due to their fairly close λ_max_ values and the noise inherent in MSP [32]. Regulatory differences are also apparent [42,44] but the high level of sequence similarity between these paralogs (~95% identical at the nucleotide level) makes quantitative PCR studies challenging. As a result of these methodological difficulties, it is sometimes simply assumed that the RH2aα and RH2aβ paralogs are sufficiently similar to justify treating them equivalently [45,46]. However, there is reason to believe that comparably small differences in λ_max_ can be ecologically and evolutionarily important in African cichlids [38], and whether or not these opsins are functionally equivalent from the perspective of African cichlid visual biology and fitness is not clear. Comparative sequence analysis may be able to provide useful insights into this system.

Given the important role vision plays in the African cichlid adaptive radiation [31,32] as well as the important role gene duplication plays in functional diversification in general [3], we set out to explore patterns of sequence evolution associated with the RH2aα-RH2aβ gene duplication event using both Branch-site and Clade codon-substitution model approaches. We document complex patterns of divergence among duplicated African cichlid RH2aα and RH2aβ opsins, reflecting both paralog-specific and species-specific processes. Importantly, positive selection was documented not using Branch-site models, but the less widely employed Clade models. We discuss the implications of our findings in light of gene duplication theory, cichlid visual ecology, and opsin structure and function.

## Methods

Phylogenetic analyses were carried out on a data set of 48 fish RH2 opsin sequences from 29 species; species names and accession numbers are provided in Additional file 1: Figure S1. Translated amino acid sequences were assembled in MEGA 4 [47] and aligned using ClustalW [48], after which the extreme N- and C-termini of the opsin sequences were trimmed, leaving an alignment 343 codons in length. Bayesian phylogenetic analysis was carried out using MrBayes 3.2 [49] using the GTR+I+Γ nucleotide substitution model, which was selected based on AIC rank, as calculated by MrModeltest 2.2 [50]. Four runs, each consisting of four chains (three heated and one cold), were run for 5 x 10^6^ generations, sampling every 100 generations. The first 25% of the samples were considered ‘burn-in’ and discarded. Adequate sampling and convergence were assured by ensuring that (1) the standard-deviation of split frequencies was less than 0.01 by the end of the analysis, (2) post-scale reduction factors were approximately 1.000 for all parameter estimates and topological partitions, (3) parameter estimate-by-generation plots were stationary, and (4) effective sample sizes were greater than 100. These checks were carried out through direct examination of the MrBayes output file and using Tracer 1.5 [51]. A codon-partitioned approach was employed as well, assuming separate GTR+I+Γ substitution models for each of the three codon positions. Maximum likelihood (ML) phylogenetic analyses were carried out using PhyML 3.0 [52] assuming the GTR+I+Γ nucleotide substitution model. Ten random trees plus a tree inferred using the BIONJ algorithm were used as starting trees, and both nearest neighbour interchange (NNI) and subtree-pruning and regrafting (SPR) tree-search approaches were used to explore tree space. Node support for the ML tree was evaluated using the SH-like approximate likelihood ratio test approach [52]. For both Bayesian and ML analyses, four rate categories were assumed for the +Γ distribution used to described among-site rate variation [53].

**Figure 1:**
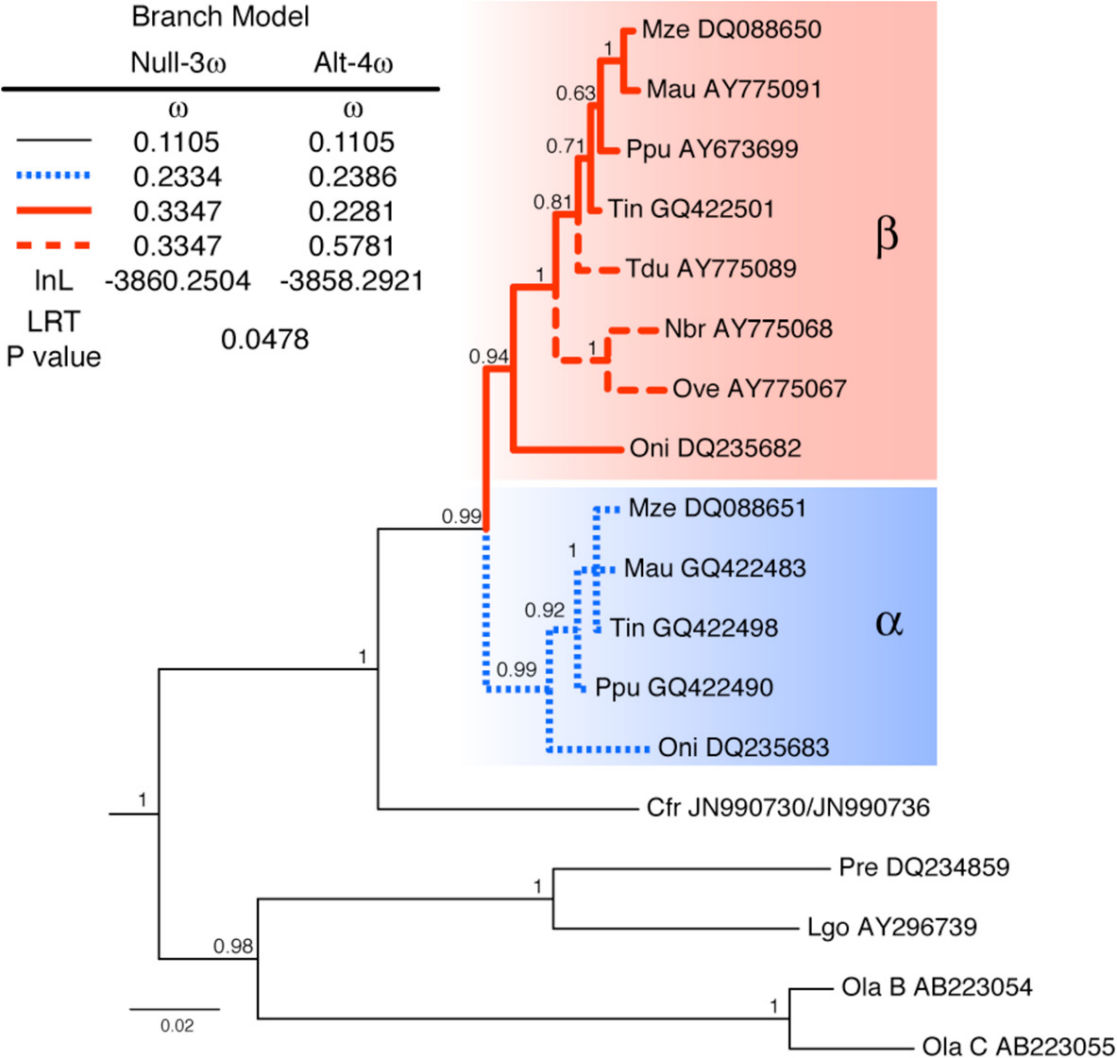
**RH2a opsin gene tree showing the African cichlid-specific duplication event that produced the RH2aα (thick, dotted, blue branches) and RH2aβ (thick, solid/dashed, red branches) opsins.** Dashed red branches indicate RH2aβ lineages from Lake Tanganyikan cichlids. Numbers adjacent to nodes indicate clade posterior probability, and branch lengths indicate expected number of substitutions per nucleotide site. Branch model analyses (inset, upper left) revealed that ω was significantly elevated along the Lake Tanganyikan branches compared to the rest of the RH2aβ clade. Species code abbreviations indicate the first letter of the genus and first two letters of each species: Cfr, *Crenicichla frenata*; Lgo, *Lucania goodei*; Mau, *Melanochromis auratus*; Mze, *Metriaclima zebra*; Nbr, *Neolamprologus brichardi*; Ola, *Oryzias latipes*; Oni, *Oreochromis niloticus*; Ove, *Ophthalmotilapia ventralis*; Ppu, *Pundamilia pundamilia*; Pre, *Poecilia reticulata*; Tdu, *Tropheus duboisi*; Tin, *Tramitichromis intermedius*. Genbank accession numbers are included alongside the species codes.

After estimating the fish RH2 phylogeny, we focused on the RH2a opsin clade for subsequent molecular evolutionary analyses. Specifically, we extracted and analyzed the monophyletic sub-tree containing the duplicated African cichlid RH2aα and RH2aβ opsins plus five outgroup RH2a sequences; species names and accession numbers for these sequences can be found in Figure 1 and Additional file 1: Figure S1. Due to uneven taxonomic coverage in online genetic databases, the RH2aβ clade possesses opsin sequences from three additional species compared to the RH2aα clade. The three extra sequences in the RH2aβ clade are all derived from Lake Tanganyikan cichlids, which were surveyed prior to the discovery of the RH2a duplication event [54]; whether these species have retained or lost their RH2aα opsins is currently not known, though current phylogenetic hypotheses for Lake Tanganyikan cichlids [55] suggest that multiple deletions would be required for all three species to truly lack RH2aα opsins in their respective genomes (see Additional file 1: Figure S2). Note that the RH2aβ sequences from *Ophthalmotilapia ventralis* and *Neolamprologous brichardi* were obtained from cDNA even though relative RH2a gene expression is skewed towards the RH2aα paralog in *Oreochromis niloticus* and Lake Malawi haplochromine cichlids [42,44]. RH2a opsin sequences are available for many other African cichlids, but often differ from one another at just one or two positions; because including all such sequences would greatly increase computational time without adding much information, we chose to focus on key sequences representing well-studied species and key cichlid lineages.

We explored patterns of selective constraint across this RH2a data set through the use of codon substitution models that include the nonsynonymous to synonymous substitution rate ratio (ω or dN/dS) as a parameter [56,57]. The ω ratio speaks to the form and strength of selective constraint operating on protein-coding DNA: 0 < ω < 1 is consistent with purifying selection (nonsynonymous substitutions are accumulating more slowly than synonymous substitutions), ω = 1 suggests neutrality (nonsynonymous and synonymous substitutions are accumulating at equivalent rates), and ω > 1 indicates positive selection (nonsynonymous substitutions are accumulating faster than synonymous substitutions). Three different approaches were used, each of which makes different assumptions about how ω varies across the alignment and/or across the phylogeny: (1) Branch models [58], (2) Branch-site models [16], and (3) Clade models [20]. Models were fit to the data using the codeml program of the PAML 4.2 software package [59]. Within each of these model classes, likelihood ratio tests (LRTs) were used to compare the fit of complex models against simpler, nested models [60,61]. LRTs were carried out by comparing twice the difference in *ln* likelihood scores of nested models against a χ^2^ distribution with the degrees of freedom equal to the number of extra parameters estimated by the more complex model. However, as LRTs can only be used to compare nested models, we also used AIC scores [62] to help convey the relative fit of the different Clade models applied to our fish RH2a data set. In addition to the selection pressure parameters (dN/dS, ω; proportions, *p*), the transition-to-transversion substitution rate ratio (κ) and branch lengths were optimized as well. Codon frequencies were approximated using the F3x4 calculation. Each model was fit to the data multiple times from different starting parameter values to help ensure local optima were avoided, with either ω or κ perturbed, as needed, depending on the particular model. As these methods assume that the aligned sequences are related by a phylogenetic tree, not by a reticulating network, the signature of gene conversion within this data set was searched for using Phi [63], as implemented in PhiPack. Significance was assessed either assuming a normally approximated null distribution or via a permutation approach (with 1000 permutations), and analyses were run with the window size left at the recommended default value of w = 100 and at w = 50. The results were qualitatively equivalent in spite of these changes; as such, only results derived using the normal approximation and w = 100 are shown. Dot plots were created using eBioX 1.5.1 [64].

Branch models [65] assume that the ω ratio varies across branches of the phylogeny (specified *a priori*) but that it is invariant across sites of the alignment; comparing complex Branch models (i.e., ones with multiple ω ratios) against simpler, nested models tests whether ω varies significantly between sections of the phylogeny. Since Branch models make the unrealistic assumption of among-site homogeneity, they often lack power to detect subtle patterns of divergence across phylogenies, and we conducted post-hoc Branch model analysis simply to help demonstrate our Clade model partitioning schemes (described below). Branch-site models and Clade models similarly allow for variation in ω among pre-specified branches of the phylogeny, but, unlike Branch models, also incorporate among-site variation in selective constraint. The signature of positive selection (ω > 1) along pre-specified lineages was tested for using the Branch-site approach of Zhang, Nielsen, and Yang [16]. This model assumes that there are four classes of sites and that the phylogeny can be divided into ‘background’ and ‘foreground’ lineages based on an *a priori* hypothesis about when positive selection may have occurred. The first two classes of sites correspond to codons that experience selection consistently across the entire phylogeny, experiencing either purifying selection (0 < ω_0_ < 1) or neutral pressure (ω_1_ = 1), respectively. The final two classes of sites correspond to codons that experience purifying or neutral selection on the background lineages, but positive selection (ω_2_ > 1) on the foreground lineage. These four site classes comprise proportions *p*_0_ (universally-purifying site class), *p*_1_ (universally-neutral site class), *p*_0_*p_2_/(1 – *p*_2_) (purifying-to-positive selection site class), and *p*_1_*p_2_/(1 – *p*_2_) (neutral-to-positive selection site class) of the total data set (where *p*_2_ = 1 – *p*_0_ – *p*_1_). The goodness-of-fit of this Branch-site model is established by comparing it via a LRT against a constrained null model where ω_2_ = 1; this LRT thus tests for the presence of positively selected sites.

The signature of divergent selective constraint across the phylogeny was tested for using the Clade model C (CmC) approach of Bielawski and Yang [20] as modified by Yang, Wong, and Nielsen [66]. In its simplest form, CmC assumes that the branches of the phylogeny can be divided into two partitions, the ‘background’ branches and the ‘foreground’ branches. CmC accommodates among-site variation in the substitution process by assuming three site classes. As with the Branch-site approach described above, the first two classes of sites correspond to codons that experience selection consistently across the entire phylogeny, experiencing either purifying selection (0 < ω_0_ < 1) or neutral pressure (ω_1_ = 1). The third site class accounts for codons that experience divergent selection pressures in different, pre-defined partitions (i.e., ω_2_ > 0 for the background branches and ω_3_ > 0 for the foreground branches). These site classes correspond to proportions *p*_0_, *p*_1_, and *p*_2_ of the total data set (where *p*_2_ = 1 – *p*_0_ – *p*_1_). Models M1a and M2a_rel, neither of which incorporates among-lineage variation in ω, were used as null models to test for the presence of divergently selected sites. M1a is the standard null model for CmC analyses [66]; M1a possesses only two site classes: one for sites subject to purifying selection (0 < ω_0_ < 1), and one for neutral sites (ω_1_ = 1). However, our previous analyses of simulated data sets revealed that the CmC versus M1a LRT is prone to false positive test results when faced with moderate among-site variation in selective constraint. We therefore also employed our newly proposed M2a_rel null model for CmC analyses [67]; M2a_rel possesses purifying and neutral site classes, like the M1a model, but also possesses a third site class under which a single ω ratio is estimated for all branches of the phylogeny (ω_2_ > 0). We checked the robustness of our results to slight changes in model framework by reanalyzing the data using the Clade model D (CmD) framework [20]; like CmC, CmD assumes three site classes with the final class modeling divergent selection among clades but, unlike CmC, no constraints are placed on the ω estimates for any of the site classes.

Yoshida et al. [21] recently extended CmC to allow for more than two tree partitions, each with a separately estimated ω ratio. We refer to this as a ‘multi-clade’ approach, and we used this approach to examine complex patterns of divergence in selection across the phylogeny by comparing such models against simpler, nested models with fewer tree partitions. Assuming a phylogeny can be partitioned into three clades (X, Y, and Z), the multi-clade approach could be used to estimate three separate ω ratios for the three tree partitions (ω_2_ > 0 for clade X, ω_3_ > 0 for clade Y, and ω_4_ > 0 for clade Z). Comparing this model against a simpler, null model with only two tree partitions (say, ω_2_ > 0 for clade X, and ω_3_ > 0 for both clade Y and clade Z) would constitute a test of whether selective constraint is equivalent in clades Y and Z (i.e., whether or not ω_3_ ≠ ω_4_). This LRT’s null model is formed by imposing a single, non-boundary constraint on the alternative model (i.e., the constraint that ω_3_ = ω_4_), reducing model size by one estimated parameter. As a result, the null distribution for this LRT should follow a χ^2^ distribution with one degree of freedom. We therefore generated simulated data sets assuming a CmC framework (with two tree partitions), and used these data sets to evaluate this multi-clade LRT’s false-positive rate.

**Figure 2:**
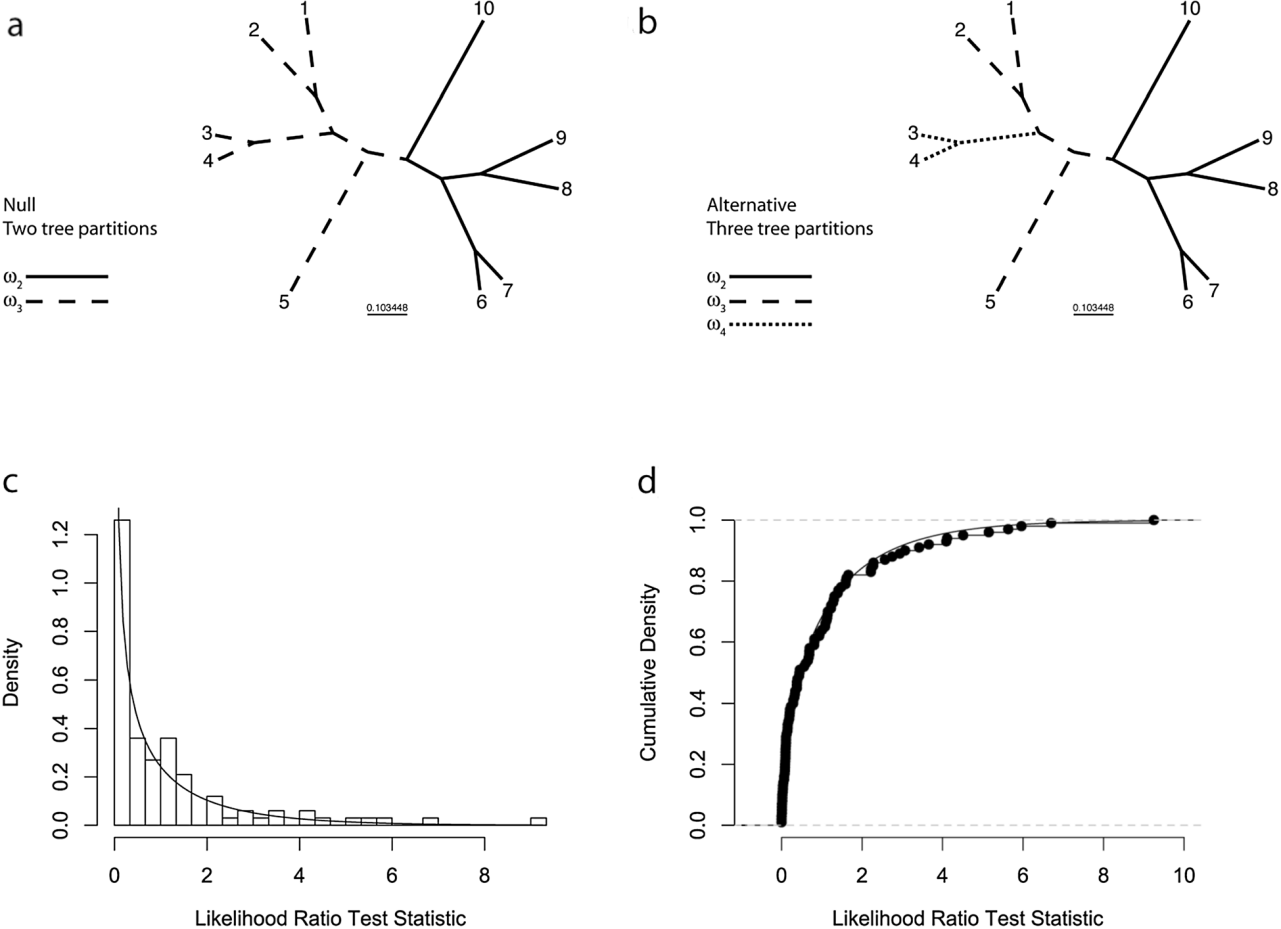
**Simulation-based analyses were used to establish the false-positive rate of the LRT comparing CmC assuming three tree partitions against a null version of CmC assuming only two tree partitions.** (**a**) The two-partition tree used for simulating null data sets. (**b**) The three-partition tree used as the LRT’s ‘alternative’ model. (**c**) Histogram showing the distribution of LRT test-statistics from analysis of 100 data sets simulated under the null model. (**d**) The same data as in (**c**), but here plotted as an empirical cumulative density function. For both (**c**) and (**d**), the solid, curved line shows the expected χ^2^_1_ distribution.

Simulated data sets were generated using the evolver program of the PAML 4.2 software package [68]. Following our earlier simulation study of CmC LRTs [67], 100 data sets of 10 taxa and 500 codons were simulated under CmC assuming the topology, branch lengths, and partitioning shown in Figure 2a. Half of the codons of each simulated data set (*p*_0_ = 0.5) were generated assuming strong purifying selection (ω = 0.0), *p*_1_ = 0.20 were generated assuming neutrality (ω_1_ = 1.0), and the remaining codons (*p*_2_ = 0.3) were simulated assuming divergent selection pressure between the solid (ω_2_ = 0.15) and dashed (ω_3_ = 0.65) tree partitions. Additional parameters for the simulations were set as follows: the transition to transversion substitution rate ratio (κ) was set to κ = 2.0; the total tree length (TL) was set to TL = 3.0 substitutions per codon; and the equilibrium frequency for each sense codon was set to 1/61. For null model analyses, we ran CmC assuming the two partitions shown in Figure 2a (i.e., correct partitioning), while for alternative model analyses, we ran CmC assuming the three partitions shown in Figure 2b (i.e., overly complex partitioning). Branch lengths were freely estimated, but κ and the equilibrium codon frequencies were fixed at their simulated values. Analyses were run multiple times from different starting ω values to help detect and avoid local optima in the likelihood surface, as described in Weadick and Chang [67]. The LRT test statistics for each of the 100 simulated data sets—twice the difference in *ln* likelihood scores from the alternative and null model analyses—were calculated, compiled, and compared against the expected χ^2^_1_ null distribution. Observed and expected cumulative density functions were compared via a one-sided Kolmogorov-Smirnov test and a one-sided binomial test. We carried out additional analyses on the 100 simulated data sets where the phylogenetic partitioning of the alternative and null models was misspecified, with branch 10 of the tree shown in Figure 2a,b included in the ‘dashed’ partition, rather than the correct ‘solid’ partition.

Following Clade model analysis, a Bayes empirical Bayes (BEB) approach was used to identify specific codons with high posterior probability (PP) of being in the ‘divergent selection’ site class [66]. BEB-identified sites were then mapped on to the three-dimensional crystal structure of bovine rhodopsin (PDB accession 1u19) [69] using MacPyMol (Delano Scientific). The phylogenetic location of specific amino acid substitutions was inferred by using ancestral reconstruction methods [70] to estimate the most probable residue at each node under the WAG+F+Γ amino acid substitution model [53,71]. Site numbering is based on alignment against bovine RH1 opsin (rhodopsin).

## Results

Phylogenetic analyses recovered reciprocally monophyletic clades of African cichlid RH2aα and RH2aβ opsins and a sister relationship between the African cichlid RH2a opsin clade and the RH2a opsin of the Neotropical cichlid *Crenicichla frenata* (Figure 1). Trees estimated via the codon-partitioned Bayesian approach and the ML approach were highly similar, and only differed with respect to the arrangement of a few of the highly similar haplochromine cichlid RH2a opsin sequences; the full Bayesian (codon-partitioned) and ML trees are provided in Additional file 1: Figure S1. The non-partitioned and codon-partitioned Bayesian methods yielded trees with equivalent branching patterns (result not shown). For molecular evolutionary analyses, we focused on the subtree corresponding to the RH2a opsins of cichlids and those of closely related atherinomorph fishes (guppy, *Poecilia reticulata*; bluefin killifish, *Lucania goodei*; and medaka, *Oryzias latipes*) from the codon-partitioned Bayesian phylogeny (Figure 1). Fitting the simple M0 codon substitution model to this data set provided an overall ω estimate of 0.1476, indicating that purifying selection is the predominant force shaping the evolution of these RH2a opsin sequences. Estimates of dS, calculated for each branch given the branch length, the number of nonsynonymous and synonymous sites in the sequence, and the overall ω estimate calculated under the M0 model, were always well below one, indicating that saturation of synonymous substitutions is unlikely to adversely affect our analyses.

Gene conversion between paralogs violates the assumptions of ML methods for estimating ω, and can even cause spurious signatures of positive selection under some conditions [72]. It has previously been suggested that gene conversion is unlikely to affect the African cichlid RH2a opsins because the paralogs are arranged in a head-to-head manner [42]. However, recent work on rodent genomes has shown that duplicates oriented in such a fashion are as prone to gene conversion as those oriented in the typical head-to-tail manner [73]. We therefore used Phi to test for local correlations in phylogenetic incompatibility across the data set (i.e., across the alignment). While we did detect a significant signature of recombination (*P* = 0.028), this reflected gene conversion between the distantly related RH2B and RH2C paralogs of the medaka (*Oryzies latipes*), not gene conversion between the African cichlid RH2a opsins. Unlike the African cichlid RH2a paralogs, these duplicated medaka opsins are oriented in a head-to-tail manner, indicating an independent duplication event [40]. Visual inspection revealed that the third and fourth introns of the medaka’s RH2B and RH2C paralogs are highly similar, while the first and second introns are divergent (Additional file 1: Figure S3), and removing either (or both) of the medaka RH2B/C paralogs from the data set eliminated the signature of conversion (*P* > 0.50 in all cases). Furthermore, strong gene conversion should result in sequences clustering by species, not by paralog, and this pattern is not observed within the African cichlid RH2a opsin portion of the estimated phylogeny (Figure 1). These results suggest that gene conversion has not had a notable impact on the evolutionary trajectories of the coding sequences of the duplicated African cichlid RH2a opsins and thus should not have adverse effects on our analyses. Visual inspection of the RH2aα and RH2aβ opsins of *Oreochromis niloticus*[74], however, revealed that introns one and four are highly similar, while introns two and three are relatively divergent (Additional file 1: Figure S3). This may indicate that natural selection is maintaining distinct opsin coding sequences in spite of at least some intronic gene conversion, as occurs in human red and green opsins [75]. Alternatively, the fact that these introns are highly similar may reflect strong purifying selection on non-coding motifs with roles in gene regulation or splicing control; more intronic RH2a sequence data, obtained from numerous species, will be needed to address these possibilities.

Several amino acid substitutions occurred along the RH2aα and RH2aβ post-duplication branches (Additional file 1: Table S1), but in neither case did we obtain evidence indicative of adaptive divergence following gene duplication using Branch-sites methods (Table 1). The Branch-site LRT for positive selection [16] was applied four times, with different branches set as the foreground partition: (1) the RH2aα post-duplication branch; (2) the RH2aβ post-duplication branch; (3) both post-duplication branches, combined; and (4) the branch joining the paralogous clades in a reduced data set for which outgroup sequences were excluded. Our Branch-site tests remained non-significant even when a more liberal null distribution (a 50:50 mixture of 0 and χ^2^_1_) was employed instead. We do note, however, that the substitutions inferred along the RH2aβ post-duplication branch were densely clustered (substitutions occurred at six sites in a 13 site stretch: sites 27–39; Additional file 1: Table S1). This region of the protein has recently been proposed to serve as the entry channel for the retinal chromophore into the protein’s binding pocket [76]. One of these substitutions (I36F) involved the replacement of a non-aromatic isoleucine residue with an aromatic phenylalanine residue at a site located at the base of the proposed entry channel; aromatic residues are thought to assist in retinal uptake [76], suggesting that this change may enhance visual pigment regeneration rate.

**Table 1:** **Parameter estimates, log-likelihood scores, and likelihood ratio test (LRT) *P *values obtained from Branch-site analyses of the RH2a data set**

**Model (n.p.)**	**Site class 0**		**Site class 1**		**Site class 2**		**κ**	***ln*L**	**LRT *P *value**
	**ω**_**0**_	***p***_**0**_	**ω**_**1**_	***p***_**1**_	**ω**_**2**_	***p***_**2**_			
BrS-A α (37)	0.0235	0.8241	1	0.1759	1.0000	0.0000	1.7670	−3789.9781	1.0000
BrS-N α (36)	0.0235	0.8241	1	0.1759	1	0.0000	1.7670	−3789.9781	
BrS-A β (37)	0.0230	0.6151	1	0.1296	1.0000	0.2553	1.7652	−3789.1087	1.0000
BrS-N β (36)	0.0230	0.6151	1	0.1296	1	0.2553	1.7652	−3789.1087	
BrS-A αβ (37)	0.0234	0.8205	1	0.1746	2.8146	0.0049	1.7670	−3789.9535	0.8643
BrS-N αβ (36)	0.0235	0.8182	1	0.1743	1	0.0076	1.7669	−3789.9681	
BrS-A αβ reduced (27)	0.0000	0.7487	1	0.2514	1.0000	0.0000	2.2643	−2200.6106	1.0000
BrS-N αβ reduced (26)	0.0000	0.7487	1	0.2514	1	0.0000	2.2643	−2200.6106	

NOTE—*n.p* number of parameters.

Given our initial findings using Branch-site methods, we explored alternative signatures of divergence in selective constraint using the Clade model C (CmC) approach [20,21,66]. First, we built on our earlier simulation-based study of CmC LRTs [67] in order to evaluate the appropriateness of the χ^2^_1_ null distribution for the multi-clade LRT comparing CmC with three tree partitions against a simpler, nested version of CmC with only two tree partitions [21]. The empirical and expected distributions of LRT test statistics from our simulation analyses are plotted in Figures 2c and 2d, and it can be seen that the two distributions follow one another quite closely. Parameter estimates under the alternative and null model are summarized in Additional file 1: Figure S4. Although eight of the 100 tests indicated positive test results, this value is not significantly different from the expected 5% (one-sided binomial test: *P* = 0.1280). Furthermore, a Kolmogorov-Smirnov test comparing the observed and expected cumulative density functions was non-significant, indicating a good fit to the expected null distribution (one-sided test for an empirical distribution falling below the χ^2^_1_ distribution: D = 0.0609; *P* = 0.4759). Our simulation results therefore suggest that the LRT comparing CmC with three tree partitions against a simpler, nested version of CmC with two tree partitions can be evaluated using a χ^2^_1_ null distribution, though we note that further analyses are needed to fully evaluate the reliability and power of this approach. Recently, Gossmann and Schmid [77] carried out similar simulation-based analyses of this LRT, concluding that it has fair-to-good power and a relatively low false positive rate; however, these analyses were carried out on smaller data sets than we employed here.

Additional analyses of the simulated data sets were carried out using an incorrect phylogenetic partitioning strategy. Given the tree shown in Figure 2b, we treated the branch leading to tip #10 as part of the ‘dashed’ partition, rather than the correct ‘solid’ partition, and we then tested for divergence (i.e., ω_3_ ≠ ω_4_) by comparing the ‘dotted’ partition against the expanded, heterogeneous ‘dashed’ partition. Based on the simulated branch lengths and ω parameter values, misspecifying the phylogenetic partitioning in this way should reduce the ω_3_ estimate (to ω_3_ ≈ 0.52) compared to the ω_4_ estimate (ω_4_ ≈ 0.65), generating a signature of divergence. We found that 23 of the 100 LRTs were significant, suggesting weak power for this test under the given conditions. Maximum likelihood estimates of the parameter values appeared to be accurate for most of the parameters (ω_0_, ω_2_, ω_3_, *p*_0_, *p*_2_), though slightly upwardly biased for the ω_4_ estimate (Additional file 1: Figure S4). Given these results, we conclude that the properly specified multi-clade LRT is statistically sound, but caution that care must be taken when designating partitions for Clade model analyses. Specifically, we recommend that partitioning choices be carefully based on external considerations, such as gene duplication theory, taxonomic sampling, or phylogenetic patterns of niche variation. Of course, additional simulation-based studies of this LRT’s properties will be beneficial, and future work should address the performance of this test using larger data sets and assuming more complex evolutionary scenarios designed to challenge the assumptions of the alternative and null models.

We first applied CmC with either the entire RH2aα clade (‘CmC α’) or the entire RH2aβ clade (‘CmC β’) set as the foreground partition (Figure 1); all other branches comprised the background partition. In both cases, the ω ratio for the divergent selection site class changed from less than one on the background lineages (ω_2_ ≈ 0.55–0.65) to greater than one on the foreground lineages (ω_3_ ≈ 1.15–1.53), with approximately 21% of the data set assigned to the divergent selection site class (Table 2). These estimates suggest that selective constraint was relaxed following duplication, and may indicate the action of weak positive selection as well. Both models fit the data significantly better than the M1a null model, but ‘CmC α’ did not fit the data significantly better than the more reliable M2a_rel null model (Table 3). However, this ‘CmC α’ model includes the RH2aβ clade as part of the ‘background’ partition alongside the outgroup orthologs of non-African cichlids. This may be inappropriate, as the ‘CmC β’ vs. M2a_rel LRT suggested a large increase in ω for the RH2aβ clade. To evaluate this possibility, we employed a multi-clade CmC approach assuming three partitions: the RH2aα branches, the RH2aβ branches, and the outgroup orthologous branches. Using this model, which we call the ‘CmC α & β’ model, it was estimated that approximately 21% of the data set evolved under divergent selective constraint across the three partitions, with a ω ratio less than one along the outgroup branches (ω_2_ = 0.50), slightly above one along the RH2aα branches (ω_3_ = 1.13), and somewhat higher still along the RH2aβ branches (ω_4_ = 1.54) (Table 2). Comparison against the ‘CmC β’ model yielded a significant LRT result (Table 3), indicating that selective constraint did indeed change after duplication in the RH2aα clade; this difference only became statistically significant once the elevated ω ratio for the RH2aβ clade was accounted for in the model. Comparison against a different null model, one where all African cichlid RH2a branches were considered as a single foreground partition (termed the ‘CmC αβ’ model), revealed that the ω ratios estimated for the RH2aα and RH2aβ partitions under the ‘CmC α & β’ model were not significantly different from one another (Table 3), with a common ω ratio estimate of ω_3_ = 1.39 (Table 2).

**Table 2:** Parameter estimates, log-likelihood scores, and AIC weights obtained from Clade model analyses of the RH2a data set

**Model (n.p.)**	**SC 0**		**SC 1**		**SC 2**		**κ**	***ln*L**	**Δ AIC**	**AIC weight**
	**ω**_**0**_	***p***_**0**_	**ω**_**1**_	***p***_**1**_	**ω**_**2**_**, ω**_**3**_**, ω**_**4**_	***p***_**2**_				
CmC αβ_MVR_ & β_T_ (39)	0.0142	0.7890	1	0.0000	ω_2_: 0.4988	0.2110	1.7071	−3789.9781	—	0.6394
					ω_3_: 1.0919					
					ω_4_: 2.6122					
CmC αβ (38)	0.0151	0.7925	1	0.0000	ω_2_: 0.5062	0.2075	1.7089	−3776.0852	2.19	0.2143
					ω_3_: 1.3889					
CmC α & β (39)	0.0147	0.7911	1	0.0000	ω_2_: 0.5037	0.2089	1.7085	−3775.7671	3.55	0.1084
					ω_3_: 1.1308					
					ω_4_: 1.5420					
CmC β (38)	0.0127	0.7838	1	0.0000	ω_2_: 0.5471	0.2163	1.7033	−3778.3671	6.75	0.0219
					ω_3_: 1.5315					
CmC β_T_ (38)	0.0115	0.7789	1	0.0000	ω_2_: 0.5882	0.2211	1.6971	−3778.6874	7.39	0.0159
					ω_3_: 2.5296					
CmC α (38)	0.0138	0.7882	1	0.0000	ω_2_: 0.6458	0.2119	1.6993	−3784.5357	19.09	0.0000
					ω_3_: 1.1498					
M2a_rel (37)	0.0089	0.7639	1	0.0512	ω_2_: 0.5401	0.1849	1.6956	−3785.9512	19.92	0.0000
M1a (35)	0.0235	0.8241	1	0.1759	-	-	1.7669	−3789.9781	23.97	0.0000

NOTE—n.p. = number of parameters; SC = site class.

Models are ranked according to AIC score.

**Table 3:** **Likelihood ratio test (LRT) *P *values for nested Clade model C (CmC) comparisons**

**Null model**	**Alternative model**					
	**M1a**	**M2a_rel**	**CmC α**	**CmC β**	**CmC αβ**	**CmC β**_T_
CmC αβ_MVR_ & β_T_	< 0.0001 (4)	< 0.0001 (2)	-	-	0.0408 (1)	0.0022 (1)
CmC α & β	< 0.0001 (4)	< 0.0001 (2)	< 0.0001 (1)	0.0226 (1)	0.4251 (1)	-
CmC αβ	< 0.0001 (3)	< 0.0001 (1)	-	-	-	-
CmC α	0.0124 (3)	0.0925 (1)	-	-	-	-
CmC β	< 0.0001 (3)	0.0001 (1)	-	-	-	-
CmC β_T_	< 0.0001 (3)	0.0001 (1)	-	-	-	-

Degrees of freedom for each LRT are indicated in parentheses.

Our RH2a data set was taxonomically unbalanced, possessing three Lake Tanganyikan cichlid RH2aβ sequences without corresponding RH2aα paralogs, and our Branch model analyses revealed that selective constraint was significantly different between the branches corresponding to these Lake Tanganyikan cichlid opsin RH2aβ lineages and the remaining RH2aβ branches (Figure 1, inset). It is therefore possible that the increased ω ratio observed for the RH2aβ clade in our CmC analyses was driven by these Lake Tanganyikan RH2aβ sequences. In lieu of removing these taxonomically-unbalancing sequences from the alignment, which would represent an unfortunate loss of data, we carried out further analyses using the recently developed multi-Clade approach [21]. Specifically, we added a third partition to the ‘CmC αβ’ model that separated the Lake Tanganyikan RH2aβ branches (β_β_) from the Lake Malawi, Lake Victoria, and riverine RH2aα and RH2aβ branches (αβ_MVR_). Using this model, which we call the ‘CmC αβ_MVR_ & β_β_’ model, it was estimated that approximately 21% of the data set evolved under divergent selective constraint, with a ω ratio less than one along the outgroup branches (ω_2_ = 0.50), slightly above one along the αβ_MVR_ branches (ω_3_ = 1.09), and substantially higher along the β_T_ branches (ω_4_ = 2.61) (Table 2). Out of the Clade models considered, the ‘CmC αβ_MVR_ & β_T_’ model was the best fitting according to AIC scores, and comparison against the ‘CmC αβ’ model yielded a significant LRT result (Table 3), indicating that the ω ratio differs significantly between the Lake Tanganyikan cichlid RH2aβ opsins (the β_T_ partition) and the RH2aα and RH2aβ opsins of other African cichlids (the αβ_MVR_ partition). Importantly, partitioning the tree in this manner did not eliminate the ω ratio increase observed for African cichlid RH2a opsin clade compared to the outgroup orthologs (LRT against ‘CmC β_T_’; Tables 2, 3). Finally, the divergent ω ratio estimate for the Lake Tanganyikan RH2aβ branches (the β_T_ partition) was significantly greater than ω = 1 (Table 4), indicating that the increase in ω seen for the β_T_ partition is due to site-specific positive selection and not simply relaxed functional constraint. Conversely, the divergent ω ratio estimated for the αβ_MVR_ partition, though elevated, was not found to significantly exceed ω = 1 (Table 4).

**Table 4:** **Likelihood ratio tests (LRTs) to determine whether foreground ω estimates from the ‘CmC αβ**_**MVR**_**& β**_**T**_**’ model significantly differ from ω = 1**

**Foreground partition**	**Unconstrained ω estimate**	***ln*L with constraint ω = 1**	***P*-value**
αβ_MVR_ (RH2aα and non-Tanganyikan RH2aβ branches)	1.0919	−3774.0580	0.7166
β_T_ (Tanganyikan RH2aβ branches)	2.6122	−3777.2771	0.0104

Both LRTs have 1 degree of freedom.

Reanalyzing the data under the CmD framework gave broadly similar results (compare Tables 2 and 3 with Additional file 1: Tables S2 and S3). First, parameter estimates were qualitatively similar between CmC and CmD analogues. Second, the rank order of CmC and CmD analogues was almost completely equivalent, with the only difference being the relative position of the two and three site-class null models (M1a and M2a_rel for CmC; M3 (*K* = 2) and M3 (*K* = 3) for CmD), which in both cases were very poor fits to the data. And, finally, *P* values for the various LRTs were generally similar, with only three tests providing qualitatively different results under the two model frameworks (one test achieved significance under CmD but not under CmC, and two others achieved significance under CmC but not CmD); notably, non-significant *P* values were still less than *P* = 0.10 for these each of these three cases. Notwithstanding these few differences, the broad similarity of AIC ranks, the majority of the LRTs, and the qualitative agreement in parameter estimates suggest that our CmC analyses have provided reliable inferences on cichlid RH2a opsin evolution.

**Figure 3:**
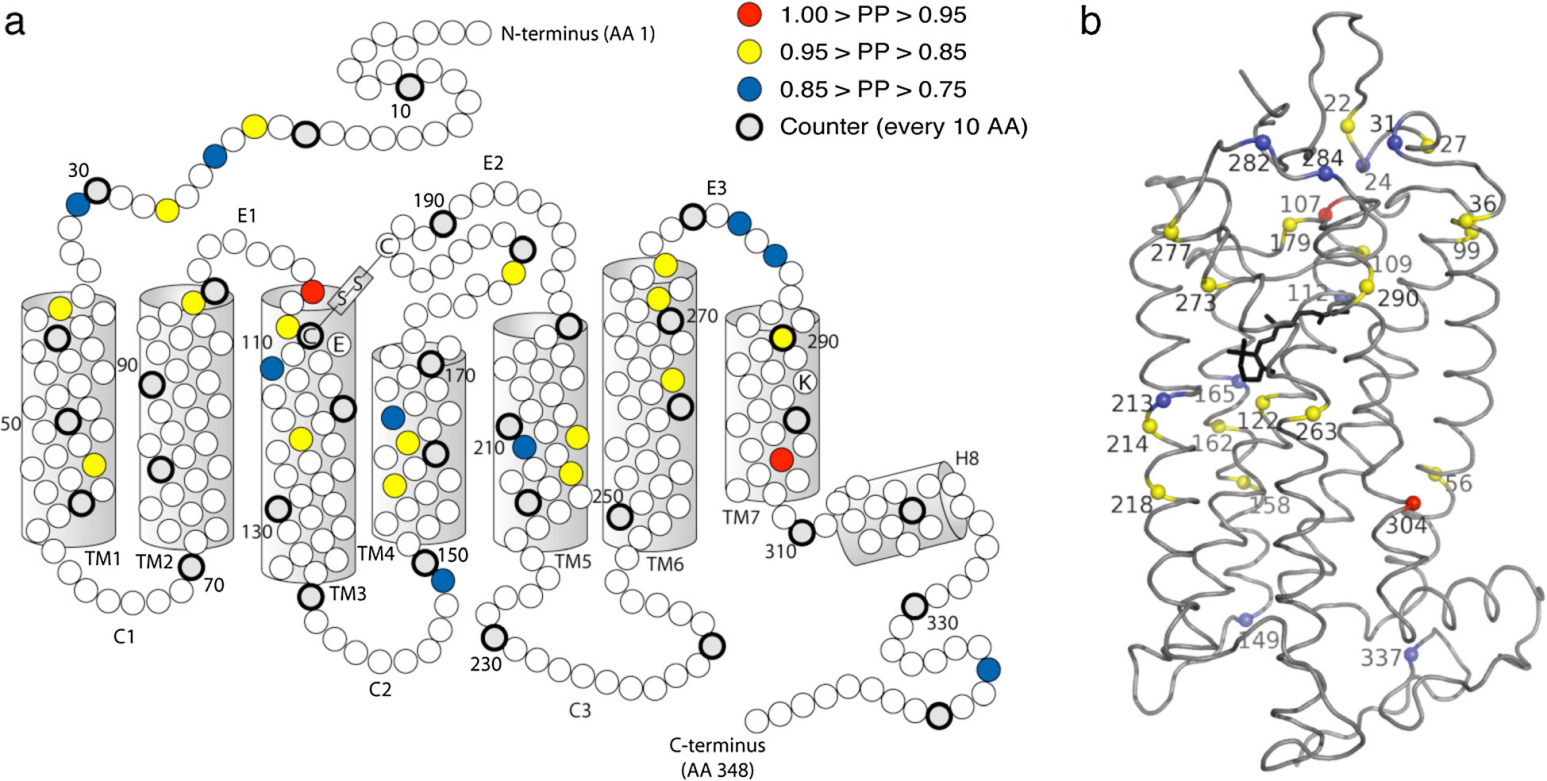
**Opsin snake plot (a) and 3D model (b) showing sites inferred to be evolving under divergent selection pressure according to Bayes empirical Bayes analysis with the CmC αβ**_**MVR**_**& β**_**T**_**Clade model.** Only sites with PP > 0.75 are shown. (**a**) The seven transmembrane α helixes (TM1-TM7), helix 8 (H8), and the extracellular (E1-E3) and cytoplasmic (C1-C3) loops are labeled. Every 10^th^ amino acid (starting with AA 10) is shaded grey and drawn in thicker stroke. (**b**) The opsin backbone is shown as a gray ribbon, while the retinal chromophore is shown in black stick format. The 3D model is viewed with the extracellular side on top. For both (**a**) and (**b**) the protein structure and numbering follow bovine rhodopsin.

Eighty-one of the 343 total codons in the alignment were variable at the amino acid level and, of these, 27 were identified by BEB analysis as members of the divergently evolving site class under the best fitting ‘CmC αβ_MVR_ & β_T_’ model (PP > 0.75 for each site) (Table 5). Included among these identified sites are seven of the 10 sites that substituted along the RH2aα and RH2aβ post-duplication branches (Additional file 1: Table S1). The position of these sites within the opsin protein's secondary and tertiary structure are shown in Figure 3. Most of these sites are situated within the opsin protein’s extracellular half (Figure 3) and several are, or are adjacent to, known RH2 pigment spectral tuning sites (Table 5). Most prominent among these is site 122; all African cichlid species in our data set possess a glutamate residue (E122) at this site except for the Lake Tanganyikan species *Ophthalmotilapia ventralis*, which instead possesses a glutamine residue (Q122). The E122Q substitution is known to have a large effect on RH2 pigment spectral sensitivity, shifting λ_max_ to shorter wavelengths by ~12-16 nm [78-80]. Moreover, this substitution is known to dramatically affect non-spectral properties of visual pigments (discussed below). Two other notable substitutions occurred along this same branch: F213V and G273V. Mutating site 273 has been shown to affect retinal uptake [76,81], while substitutions at site 213 are known to have slight effects on λ_max_ in zebrafish (*Danio rerio*) RH2 opsins [80]. Furthermore, substitutions at site 213 and 273 have been experimentally shown to influence the decay rate of the activated visual pigment in zebrafish RH1 opsins (J.M. Morrow and B.S.W. Chang, unpublished data). Interestingly, these three sites (122, 213, and 273) all substituted independently along the branch terminating with the *Oryzies latipes* (medaka) RH2C sequence, which suggests that some non-spectral opsin properties may be evolving convergently.

**Table 5:** **Sites identified as divergently evolving by Bayes empirical Bayes inference under the ‘CmC αβ**_**MVR**_**& β**_**T**_**’ Clade model**

**Site**	**PP**	**Location**	**Notes on Opsin Structure–Function**
22	0.90	N-term	
24	0.81	N-term	
27	0.91	N-term	
31	0.75	N-term	
36	0.86	N-term / TM1	RH2 spectral tuning site [80]. Situated on edge of retinal uptake/release channel [76].
56	0.90	TM1	
99	0.91	TM2	
107	0.96	E1	Adjacent to RH2 spectral tuning site (site 108) [80].
109	0.86	E1 / TM3	Adjacent to RH2 spectral tuning site (site 108) [80]. Adjacent to cysteine bond site (site 110) [82].
112	0.80	TM3	RH2 spectral tuning site [80]. Adjacent to opsin counterion (site 113) [83].
122	0.85	TM3	Major RH2 spectral tuning site [78]. Also influences G protein activation efficiency, active-state decay rate, and visual pigment regeneration rate [84].
149	0.77	C2 / TM4	Possible phosphorylation site [85,86].
158	0.86	TM4	
162	0.94	TM4	
165	0.79	TM4	Possible RH2 spectral tuning site [80].
179	0.88	E2	
213	0.75	TM5	RH2 spectral tuning site [80].
214	0.89	TM5	Adjacent to RH2 spectral tuning site (site 213) [80].
218	0.86	TM5	RH2 spectral tuning site [80].
263	0.90	TM6	
273	0.85	TM6	Role in retinal uptake [76,81].
277	0.90	TM6 / E3	
282	0.80	E3	
284	0.75	E3	
290	0.93	TM7	
304	0.97	TM7	
335	0.79	C-term	Possible phosphorylation site [85,86].

NOTE— Site numbering follows bovine RH1 opsin. Approximate location follows Sakmar et al. [34]. Abbreviations: *N-term* N-terminal tail, *TM* Transmembrane helix, *E*, Extracellular loop, *C* Cytoplasmic loop, *C-term* C-terminal tail.

Only sites with posterior probability (PP) > 0.75 are shown. Underlined sites are those which substituted along branches within the Lake Tanganyikan cichlid RH2aβ partition of the phylogeny.

**Figure 4:**
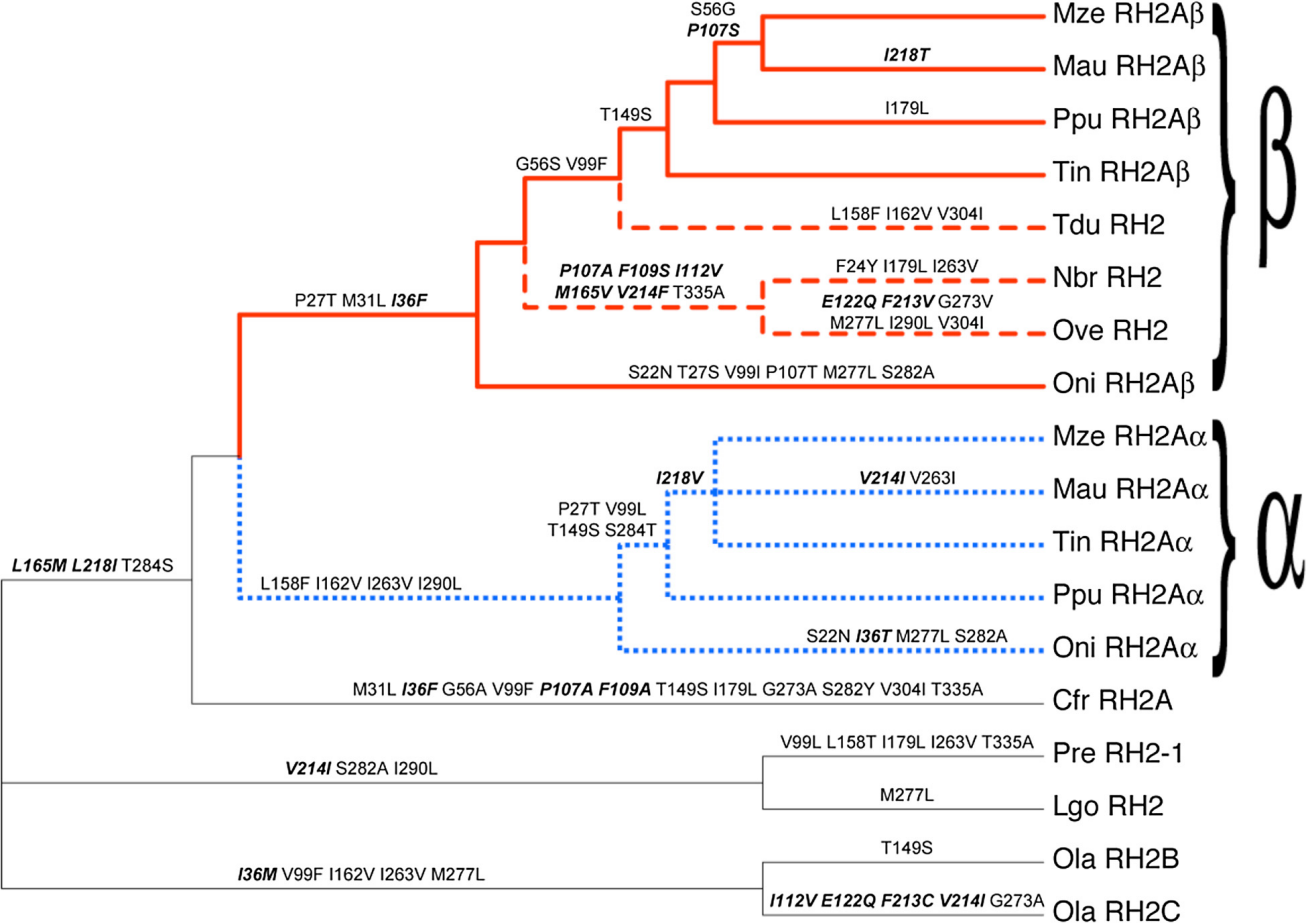
**RH2a cladogram showing the inferred location of amino acid substitutions at the 27 ‘divergently evolving’ sites listed in Table**5**.** Posterior probabilities of inferred ancestral amino acids are provided in Additional file 1: Table S4. Lines are drawn to indicate the RH2aα (dotted blue branches), RH2aβ (thick red branches), and Lake Tanganyikan tree RH2aβ partitions (dashed red branches), as in Figure 1.

A total of 178.5 amino acid changes were estimated to have occurred across the entire tree (given an alignment of 343 codons and a total tree length of 0.52032 amino acid substitutions per site, as estimated under the WAG+F+Γ amino acid substitution model). By reconstructing the ancestral amino acid residues at each node, we infer that 86 nonsynonymous changes, at minimum, occurred at the 27 BEB identified sites (mean ± SD = 3.19 ± 1.11 amino acid changes per BEB site; range = 2–6). For 18 of these 27 BEB sites, substitutions occurred along branches within the Lake Tanganyikan cichlid RH2aβ partition. Examining the changes at these sites in the context of the phylogeny reveals a few interesting patterns (Figure 4, Additional file 1: Table S4). First, BEB identified sites 273 and 277 both substituted along the branch terminating in the *Ophthalmotilapia ventralis* RH2aβ opsin sequence; these two sites are situated approximately one α helical turn apart in the opsin’s sixth transmembrane α helix, suggesting coevolutionary change. Possibly, this pattern reflects compensatory evolution, as one of the substitutions (G273V) introduced a larger amino acid while the other (M277L) introduced a smaller one. Similarly, BEB identified sites 158 and 162 are both situated one α helical turn apart in the fourth transmembrane α helix, and both substituted along the branch terminating in the *Tropheus duboisi* RH2aβ opsin sequence. Again, we see one substitution introduce a larger reside (L158F) and the other introduce a smaller residue (I162V). These two sites both project outwards into a proposed opsin dimerization interface [87], and position 162 has been experimentally shown to contribute to dimerization in the homologous dopamine D2 receptor proteins [88]. Changes at these sites may thus influence dimerization strength, which, in turn, can influence G protein binding [87]. Interestingly, these changes also occurred along the RH2aα post-duplication branch, suggesting not simply coevolution, but convergence as well. Finally, a cluster of BEB identified sites (sites 107, 109, and 112) found at the boundary between the opsin’s first extracellular loop and third transmembrane α helix all substituted along the branch leading to the last common ancestor of the *Ophthalmotilapia ventralis* and *Neolamprologus brichardi* RH2aβ opsin sequences. Site 112 is a known RH2 pigment spectral tuning site [80], while sites 107 and 109 surround another known RH2 spectral tuning site (site 108) [80]. The substitution at site 109 is particularly interesting, as the inferred change (F109S) is quite physicochemically severe, replacing a bulky, hydrophobic phenylalanine residue with a smaller, hydroxyl-bearing serine residue. More broadly, it can be seen that more sites known to affect spectral sensitivity (or that are adjacent to such sites) substitute within the RH2aβ clade compared to the RH2aα clade. Most of this difference stems from changes along the branch ancestral to *Ophthalmotilapia ventralis* and *Neolamprologus brichardi*, and along the branch terminating in *Ophthalmotilapia ventralis*.

## Discussion

Evolution after gene duplication is often characterized by some combination of relaxed selective constraint and the positively selected fixation of advantageous mutations, ultimately resulting in functional divergence among paralogs [3-5,89]. Consistent with these expectations, our results (1) revealed a dramatic change in selective constraint following the African cichlid RH2a opsin duplication event and (2) identified the signature of divergent evolution at several amino acid sites of known functional importance in RH2 visual pigments. Clade model analyses revealed that, after duplication, the selective regime experienced by many alignment sites changed from weak purifying selection to either neutral evolution or weak positive selection. Interestingly, this switch in constraint applied to both duplicated clades relative to the outgroup orthologs, and this pattern was only detected once divergence among entire clades was considered but not when just the branches immediately following the duplication event were considered.

While the patterns of evolution we observed in the fish RH2a opsin data set are consistent with a dramatic change in selective constraint following the African cichlid RH2a opsin duplication event, it is not obvious which models of gene duplication are operating here, as the observed patterns do not neatly fit the predictions of most models. The adaptive and non-adaptive (Dykhuizen-Hartl) neofunctionalization models [3,90] both posit that one copy accumulates previously deleterious substitutions, potentially leading to the evolution of a new function, while the other retains the ancestral function under a regime of purifying selection. These neofunctionalization models thus predict asymmetrical rates of evolution after duplication between paralogs, but our results indicate approximately equal shifts in selective constraint after duplication in both duplicates (with ω changing from ω ≈ 0.5 before duplication to ω ≈ 1.1–1.5, afterwards). The segregation avoidance model [3,91] proposes that duplication may beneficially fix both alleles at loci harbouring balanced polymorphisms, thus eliminating costs associated with segregation load. This model predicts that functional divergence occurs among alleles before duplication, not long after the duplicate loci have fixed as we have found. The dosage model operates when possessing multiple loci provides a beneficial increase in gene product [3,92]; this model seems inappropriate as well, as RH2aβ opsin expression is generally quite low in both *Oreochromis niloticus* and Lake Malawi haplochromine cichlids [42,44], and as the paralogs are known to be functionally divergent, contrary to model predictions. The popular duplication-degeneration-complementation model applies to multifunctional proteins, and describes a scenario by which each daughter protein neutrally loses one or more of the multiple functions such that both copies are then needed to perform all tasks [6]. This model could explain increases in ω seen in both daughter clades, but this model seems unlikely to apply to opsins, at least at the protein coding level, as it is difficult to conceive of a way opsin protein biochemistry could be subfunctionalized. Opsin proteins have several measurable biochemical phenotypes (including λ_max_, active state stability, and regeneration rate), but proper visual pigment functioning requires an integrated protein for successful phototransduction. Subfunctionalization could occur at the regulatory level, however [93].

The ‘gene sharing’ model (also referred to as the ‘specialization’ or ‘escape from adaptive conflict’ model) [7,8] seems to be the most appropriate model for our cichlid opsin data set. If a single-copy protein’s ability to efficiently serve two roles is compromised due to pleiotropy then, after duplication, each copy can adaptively specialize on one of the two roles (assuming both roles are suboptimal in the ancestor). The post-duplication ω increases we observed for our RH2a data set may indicate weak positive selection in both paralogs, which would be consistent with this prediction. While this model is typically applied to multifunctional proteins that carry out two totally different roles (e.g., a structural role and an enzymatic role, as in α lens crystallins), it can also apply to proteins that perform a single biochemical task (e.g., a particular enzymatic reaction) if there is a benefit to having copies with subtly different rates or efficiencies [4]. With regard to opsins, this could mean that a property such as λ_max_ diverged in both copies compared to the ancestor, one to a longer wavelength and one to a shorter wavelength. Similarly, structural constraints may prevent co-optimization of different aspects of the protein’s overall function. Such predictions are testable through ancestral reconstruction and functional characterization of the single-copy ancestor. Of course, it should be noted that many of these models of gene duplicate retention and evolution can act in concert or subsequent to one another [94]. Indeed, the patterns of amino acid substitutions inferred along the RH2aβ post-duplication branch are quite different than those inferred along the RH2aα post-duplication branch, with six highly-clustered sites substituting along the RH2aβ branch (Additional file 1: Table S1). Changes at these sites could conceivably influence pigment regeneration rate [76], and this may indicate that both neofunctionalization and specialization occurred following the RH2a duplication event in this system. Furthermore, here we have only considered the evolution of protein biochemistry, but these models of duplicate gene evolution can also be considered with regard to gene expression patterns.

The fact that we uncovered among-lineage ω variation when Clade models were used, but not when Branch-site models were used, opens the possibility that divergence among African cichlid RH2a opsins is also influenced by a collection of lineage-specific processes. Consistent with this hypothesis, we documented a large increase in ω along Lake Tanganyikan cichlid RH2aβ opsin branches that was above and beyond the increase already described for the RH2aβ clade. The estimated ω ratio for this tree partition was significantly above one, clearly indicating the action of positive selection. This finding is of note given that our initial focus was only on divergence associated with gene duplication, not divergence among orthologs. Our study thus serves as an example of how the evolution of duplicated genes can be driven by both paralog-specific and species-specific processes [11]. This point has practical importance as well. Many studies within the field of visual ecology assume functional equivalence among ortholgous pigments and model focal species’ perceptual abilities using data from close relatives [95]. Our results suggest that such an approach should only be applied tentatively for studies on cichlid visual ecology.

At this point, it is difficult to say what factors are behind the positive selection operating along the Lake Tanganyikan cichlid RH2aβ opsin lineages, as the visual niches inhabited by the three Lake Tanganyikan species included in our data set have surely evolved over the time-scale captured by our phylogeny. These three species inhabit distinct visual niches, varying in colour patterning, habitat depth, and diet [96], and these ecological differences could precipitate divergent selection on opsin biochemistry and expression; the detection of divergent sexually selected courtship signals or food sources may select for divergence in λ_max_, while vision under brighter or dimmer conditions could select for divergence in non-spectral, kinetic properties of visual pigments. Interestingly, some of the sites identified as positively selected along Lake Tanganyikan RH2aβ opsin lineages are known to effect both spectral (i.e., λ_max_) and non-spectral attributes of visual pigments (Table 5). Most notably, the E122Q substitution, which occurred along the terminal branch leading to the Lake Tanganyikan cichlid *Ophthalmotilapia ventralis*, is known to increase the λ_max_ of RH2 pigments by a large amount (~12-16 nm) [78-80], and to affect a number of non-spectral, kinetic properties, including the photoactivated pigment’s decay rate, the efficiency with which the activated pigment activates the downstream G protein, and the rate of visual pigment reformation following retinal release [84,97]. For each of these properties, experimentally adding the E122Q substitution to rod pigments produces mutant pigments that behave in a more cone-like manner (i.e., with a faster rate of active state decay and faster pigment regeneration). *Ophthalmotilapia ventralis* is known to generally reside at shallower depths than the other two Lake Tanganyikan cichlids in our data set (approximate depth range: *O. ventralis* 2–10 m; *N. brichardi* 5–30 m, *T. duboisi* 3–15 m) [96], where the visual environment is expected to be somewhat brighter. We thus see a compatible pattern between opsin molecular evolution and comparative visual ecology in this system. Overall, our results suggest that the RH2aβ opsins play an important role in vision in at least some Lake Tanganyikan species, despite the fact that the RH2aβ opsin has generally been found to be lowly expressed in other African cichlids.

It is notable that we were only able to uncover among-lineage ω variation once Clade models were employed, but not when the more popular Branch-site models were used. A number of factors are expected to affect the power of the Branch-site test [98], and it may be that future analyses of larger data sets will yield different results, though we suspect otherwise, as substitutions occurred at several amino acids along both of the post-duplication branches. It appears that functional divergence simply occurred in a manner undetectable by the Branch-site methods. Branch-site models assume a very specific form of functional divergence—that is, a punctuated burst of adaptive sequence turnover—but functional divergence can instead manifest as variation in the overall strength of constraint or residue conservation between clades [99]. Most of the sites that substituted along the RH2aα and RH2aα post-duplication branches also substituted along other branches of the phylogeny, and such substitution patterns may not fit neatly within the site classes defined by the Branch-site models. The design of Clade models makes them better able to detect this alternative signature of functional divergence. Furthermore, the patterns we uncovered were only detectable because the Clade model approach was recently expanded to allow for multiple foreground partitions [21], which allowed us to fit models that accommodate multiple shifts in the ω ratio across the phylogeny. To date, only three other studies have employed this new approach: Yoshida et al. [21] uncovered variation in the strength of positive selection affecting HIV *env* genes sampled from different decades; Wei and Ge [100] documented divergent selective constraint among duplicated MADS-box transcription factors in grasses; and, finally, Gossmann and Schmid [77] studied genome-wide patterns of divergence among duplicated *Arabidopsis thaliana* genes. We note that our study is the first, to our knowledge, to explicitly investigate divergence among both orthologs and paralogs in the same data set. Finally, it is noteworthy that several studies have used the results of Clade model analyses to support arguments of positive selection [101,102], but whether or not the relevant ω estimates significantly exceed ω = 1 has not been explicitly tested, as we have done here; this point, while at first glance technical in nature, is of large importance, as ω estimates larger than ω = 1 may occur due to chance.

In conclusion, our results are indicative of functional divergence among African cichlid RH2a opsins driven, at least in part, by positive selection. Combined with the insights of past studies, which indicate biochemical and expression differences among paralogs and among species, our results suggest that there is much to be learned by distinguishing among African cichlid RH2a opsin sequences, rather than grouping them together on account of their high sequence similarity. Furthermore, these results provide a framework for mechanistic studies of functional diversification among cichlid RH2a opsins, and help establish African cichlid RH2a opsins as a useful system for research on how function and linkage shape the evolution of young tandem duplicates [103]. Finally, our study adds to a growing body of research directed towards uncovering the molecular signature of diversification within the rapidly speciating African cichlid clade.

## Competing interests

The authors declare no competing interests, financial or otherwise, regarding this manuscript.

## Authors’ contributions

CJW conceived of the study, performed the analyses; CJW and BSWC designed the analyses, and wrote the paper. Both authors read and approved the final manuscript.

## Supplementary Material

Additional file 1 Supplemental Material. Click here for file
