# Supplementary material for: Complex patterns of divergence among green-sensitive (RH2a) African cichlid opsins revealed by Clade model analyses
Source: BMC Evol Biol. 2012 Oct 18;12:206. doi: 10.1186/1471-2148-12-206 (PMC3514295; doi:10.1186/1471-2148-12-206)
Supplement: Additional file 1 — Supplemental Material. [file 1471-2148-12-206-S1.pdf]

Supplemental Material

Supplementary Figure 1 (following page)—Codon-partitioned Bayesian (top) and maximum likelihood (bottom) estimates of the fish RH2 opsin phylogeny. Clade support values (posterior probabilities, top tree; aLRT values, bottom tree) are provided. Branch lengths are in estimated number of substitutions per site. The subtree subjected to molecular evolutionary analyses is shaded in gray. Sequences are indicated by three-letter species code (see below), gene identifier, and NCBI Genbank Accession number. Species codes: Abu, *Acanthopagrus butcheri*; Cfr, *Crenicichla frenata*; Dma, *Dissostichus mawsoni*; Dre, *Danio rerio*; Gac, *Gymnodraco acuticeps*; Gmo, *Gadus morhua*; Hhi, *Hippoglossus hippoglossus*; Lgo, *Lucania goodei*; Mau, *Melanochromis auratus*; Msu, *Mullus surmuletus*; Mze, *Metriaclima zebra*; Nan, *Notothenia angustata*; Nbr, *Neolamprologus brichardi*; Ola, *Oryzias latipes*; Omy, *Oncorhynchus mykiss*; Oni, *Oreochromis niloticus*; Ove, *Ophthalmotilapia ventralis*; Pal, *Plecoglossus altivelis*; Pam, *Pseudopleuronectes americanus*; Pbo, *Pagothenia borchgrevinki*; Pmi, *Pomatoschistus minutus*; Ppu, *Pundamilia pundamilia*; Pre, *Poecilia reticulata*; San, *Scopelarchus analis*; Ssa, *Salmo salar*; Tdu, *Tropheus duboisi*; Tin, *Tramitichromis intermedius*; Tni, *Tetraodon nigroviridis*; Tru, *Takifugu rubripes*.

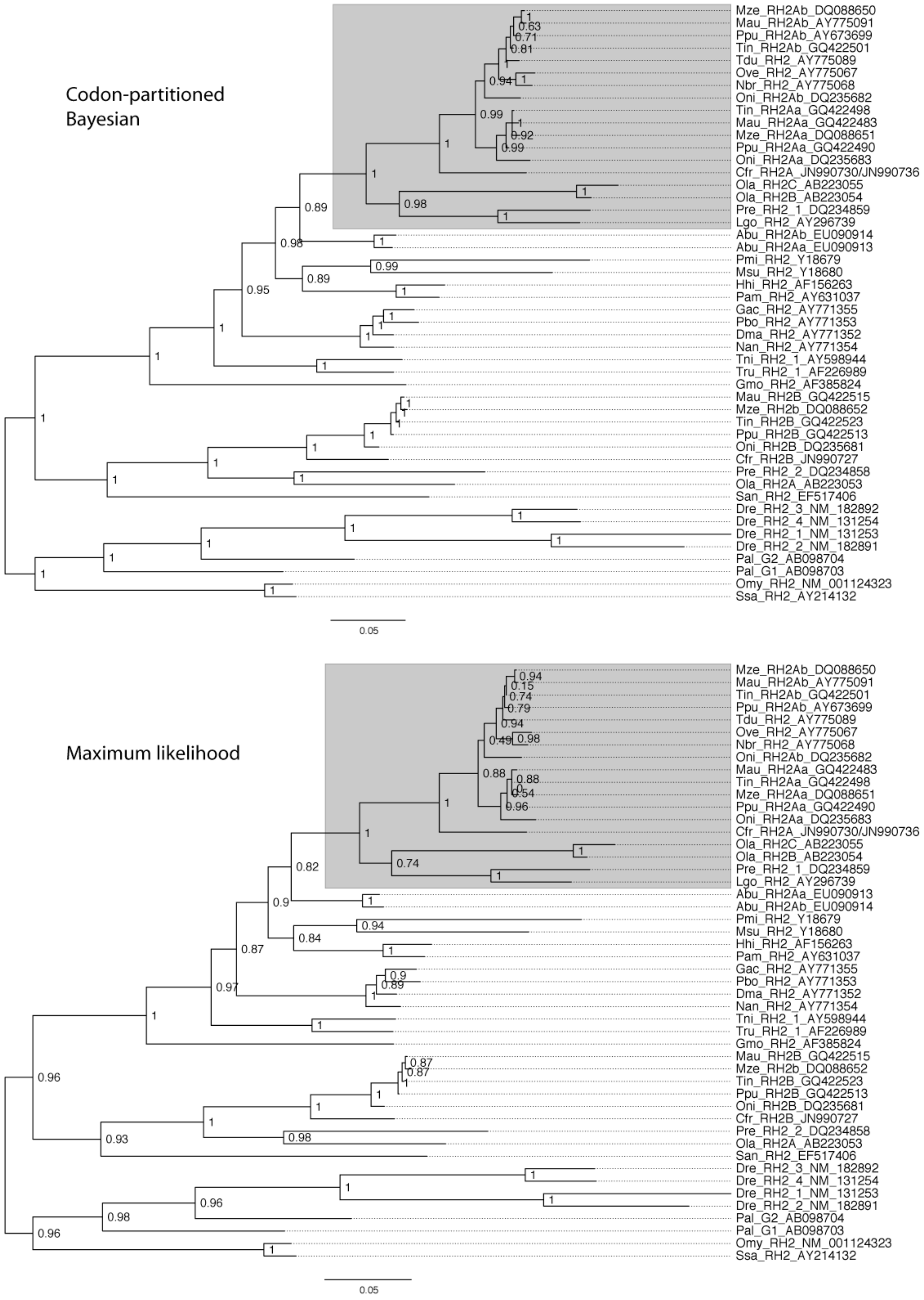
Codon-partitioned Bayesian
Mze_RH2Ab_DQ088650
Mau_RH2Ab_AY775091
Ppu_RH2Ab_AY673699
Tin_RH2Ab_GQ422501
Tdu_RH2_AY775089
Ove_RH2_AY775067
Nbr_RH2_AY775068
Oni_RH2Ab_DQ235682
Tin_RH2Aa_GQ422498
Mau_RH2Aa_GQ422483
Mze_RH2Aa_DQ088651
Ppu_RH2Aa_GQ422490
Oni_RH2Aa_DQ235683
Cfr_RH2A_JN990730/JN990736
Ola_RH2C_AB223055
Ola_RH2B_AB223054
Pre_RH2_1_DQ234859
Lgo_RH2_AY296739
Abu_RH2Ab_EU090914
Abu_RH2Aa_EU090913
Pmi_RH2_Y18679
Msu_RH2_Y18680
Hhi_RH2_AF156263
Pam_RH2_AY631037
Gac_RH2_AY771355
Pbo_RH2_AY771353
Dma_RH2_AY771352
Nan_RH2_AY771354
Tni_RH2_1_AY598944
Tru_RH2_1_AF226989
Gmo_RH2_AF385824
Mau_RH2B_GQ422515
Mze_RH2b_DQ088652
Tin_RH2B_GQ422523
Ppu_RH2B_GQ422513
Oni_RH2B_DQ235681
Cfr_RH2B_JN990727
Pre_RH2_2_DQ234858
Ola_RH2A_AB223053
San_RH2_EF517406
Dre_RH2_3_NM_182892
Dre_RH2_4_NM_131254
Dre_RH2_1_NM_131253
Dre_RH2_2_NM_182891
Pal_G2_AB098704
Pal_G1_AB098703
Omy_RH2_NM_001124323
Ssa_RH2_AY214132
0.05
Maximum likelihood
Mze_RH2Ab_DQ088650
Mau_RH2Ab_AY775091
Tin_RH2Ab_GQ422501
Ppu_RH2Ab_AY673699
Tdu_RH2_AY775089
Ove_RH2_AY775067
Nbr_RH2_AY775068
Oni_RH2Ab_DQ235682
Mau_RH2Aa_GQ422483
Tin_RH2Aa_GQ422498
Mze_RH2Aa_DQ088651
Ppu_RH2Aa_GQ422490
Oni_RH2Aa_DQ235683
Cfr_RH2A_JN990730/JN990736
Ola_RH2C_AB223055
Ola_RH2B_AB223054
Pre_RH2_1_DQ234859
Lgo_RH2_AY296739
Abu_RH2Aa_EU090913
Abu_RH2Ab_EU090914
Pmi_RH2_Y18679
Msu_RH2_Y18680
Hhi_RH2_AF156263
Pam_RH2_AY631037
Gac_RH2_AY771355
Pbo_RH2_AY771353
Dma_RH2_AY771352
Nan_RH2_AY771354
Tni_RH2_1_AY598944
Tru_RH2_1_AF226989
Gmo_RH2_AF385824
Mau_RH2B_GQ422515
Mze_RH2b_DQ088652
Tin_RH2B_GQ422523
Ppu_RH2B_GQ422513
Oni_RH2B_DQ235681
Cfr_RH2B_JN990727
Pre_RH2_2_DQ234858
Ola_RH2A_AB223053
San_RH2_EF517406
Dre_RH2_3_NM_182892
Dre_RH2_4_NM_131254
Dre_RH2_1_NM_131253
Dre_RH2_2_NM_182891
Pal_G2_AB098704
Pal_G1_AB098703
Omy_RH2_NM_001124323
Ssa_RH2_AY214132
0.05

Supplementary Figure 2—Species tree of sampled cichlid species (following Koblmuller et al. 2008 Hydrobiologia 615:5-20; Koblmuller et al. 2008 Molecular Phylogenetics and Evolution 49:153-169). Black bars indicate the likely phylogenetic location of the RH2aα/β duplication event (after the Neotropical/African cichlid split) and the missing Lake Tanganyikan cichlid RH2aβ sequence data, which, if truly reflective of genome presence/absence, may indicate one or more gene losses. Three letter species codes are defined in the caption to Figure 1.

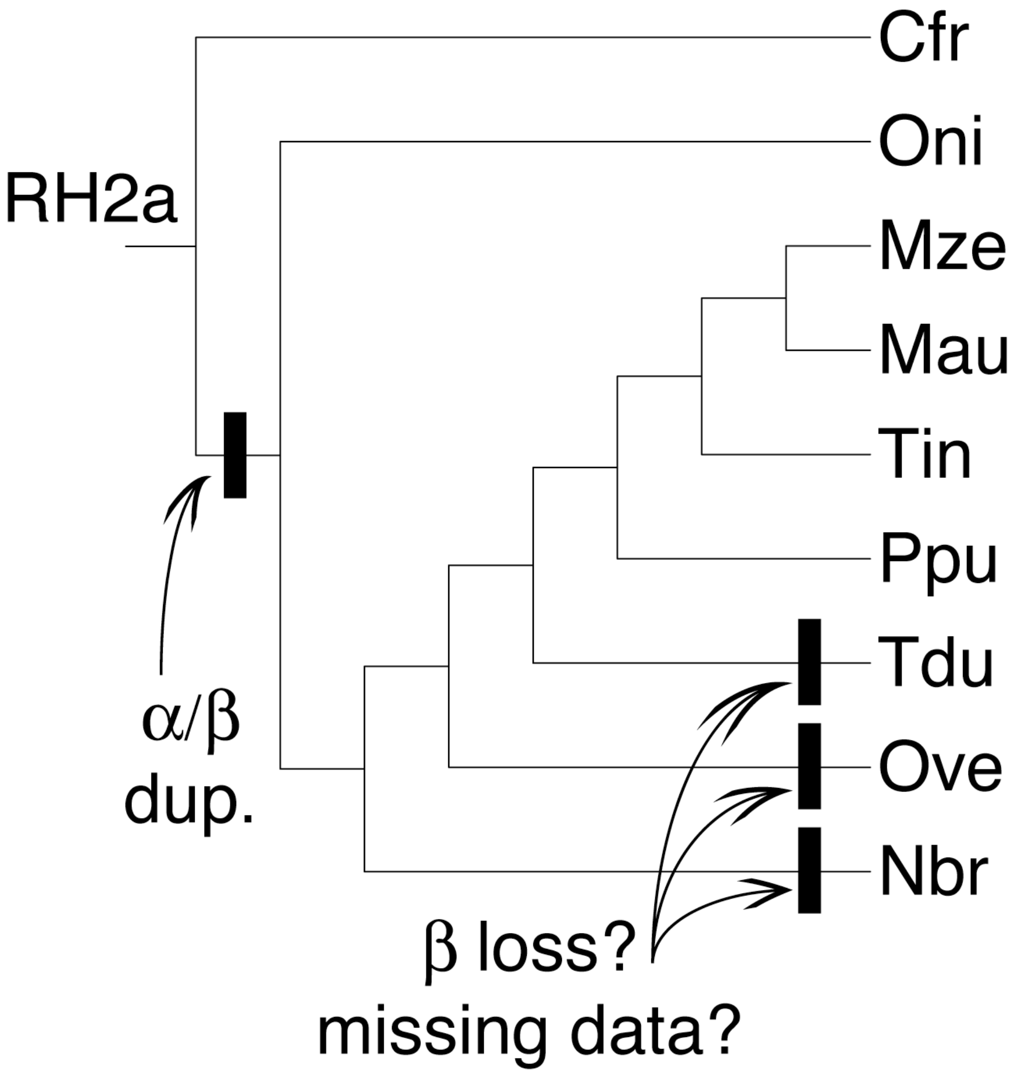

Supplementary Figure 3 (begins following page)—Dot plots visually demonstrating gene conversion between *Oryzies latipes* RH2B and RH2C paralogs (top) and between *Oreochromis niloticus* RH2aα and RH2aβ paralogs (bottom). In both cases, four sequences are compared: genomic sequence data for the two paralogs (including exons and introns) and exonic coding sequence data for the two paralogs. Dot plots were created in eBioX 1.5.1 using a window size of w = 10. In the case of the *Oryzies latipes* RH2B and RH2C sequences, the third and fourth introns are identical between paralogs, each with no indels and no sequence differences, while the first and second introns are highly divergent between paralogs, each with multiple indels and many sequence differences (intron 1 p-distance = 42%; intron 2 p-distance = 52%). In the case of the *Oreochromis niloticus* RH2aα and RH2aβ sequences, the first and fourth introns are identical in length between paralogs, each with no indels and few sequence differences (intron 1 p-distance = 2%; intron 4 p-distance = 5%), while the second and third introns are highly divergent between paralogs, each with multiple indels and many sequence differences (intron 2 p-distance = 58%; intron 3 p-distance = 36%).

Oryzies latipes RH2B / RH2C Dot Plots

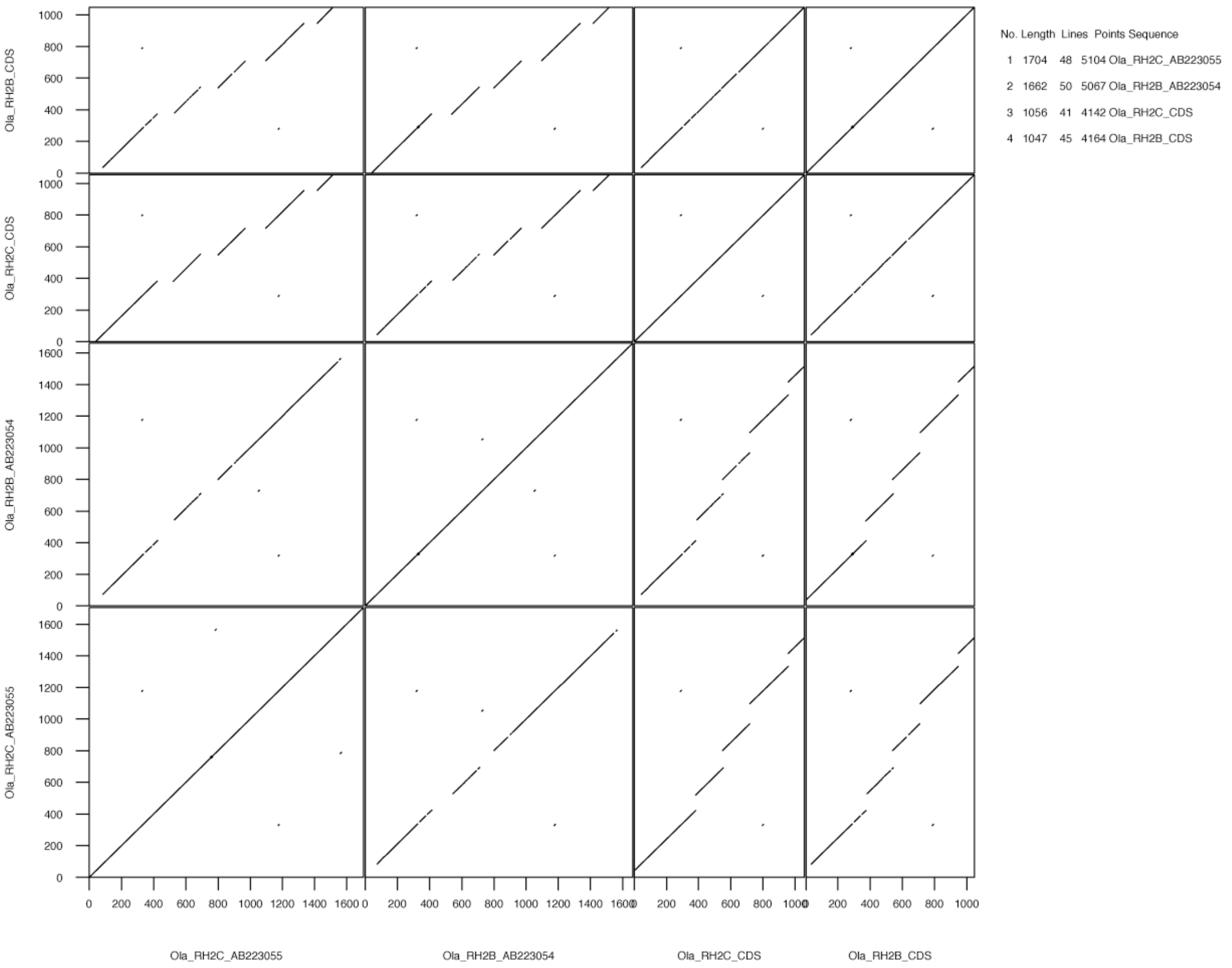

Oreochromis niloticus RH2aα / RH2aβ Dot Plots

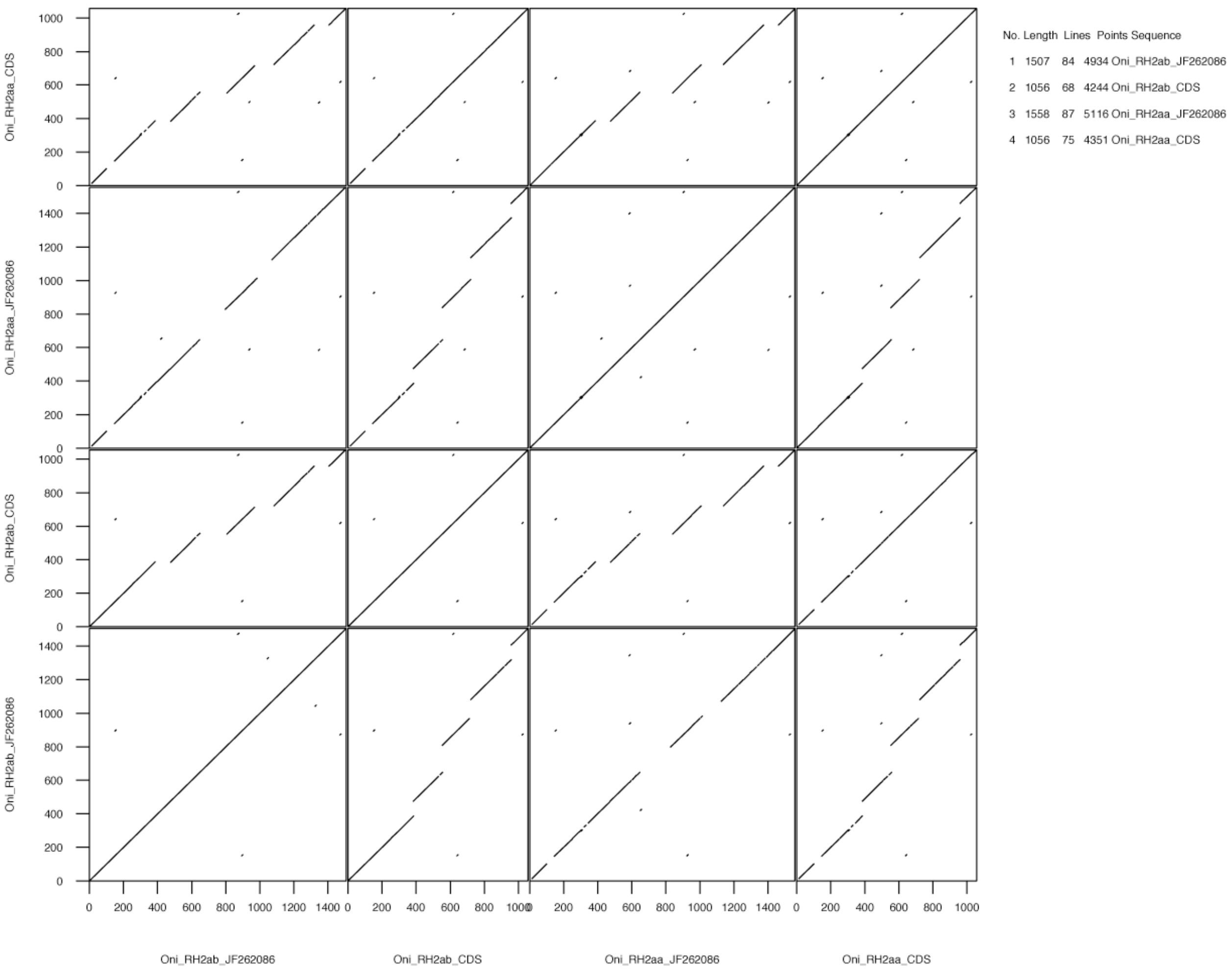

Supplementary Figure 4—Box/violin-plots displaying the distribution of maximum likelihood parameter estimates derived from Clade model C (CmC) analysis of $N = 100$ simulated data. (a) CmC-multiclade with correct partitioning. (b) CmC with correct partitioning. (c) CmC-multiclade with misspecified partitioning. (d) CmC with misspecified partitioning. The black diamonds within the box/violin plots indicate median estimates, and the arrowheads adjacent to the box/violin plots indicate the ‘true’ (simulated) parameter values.

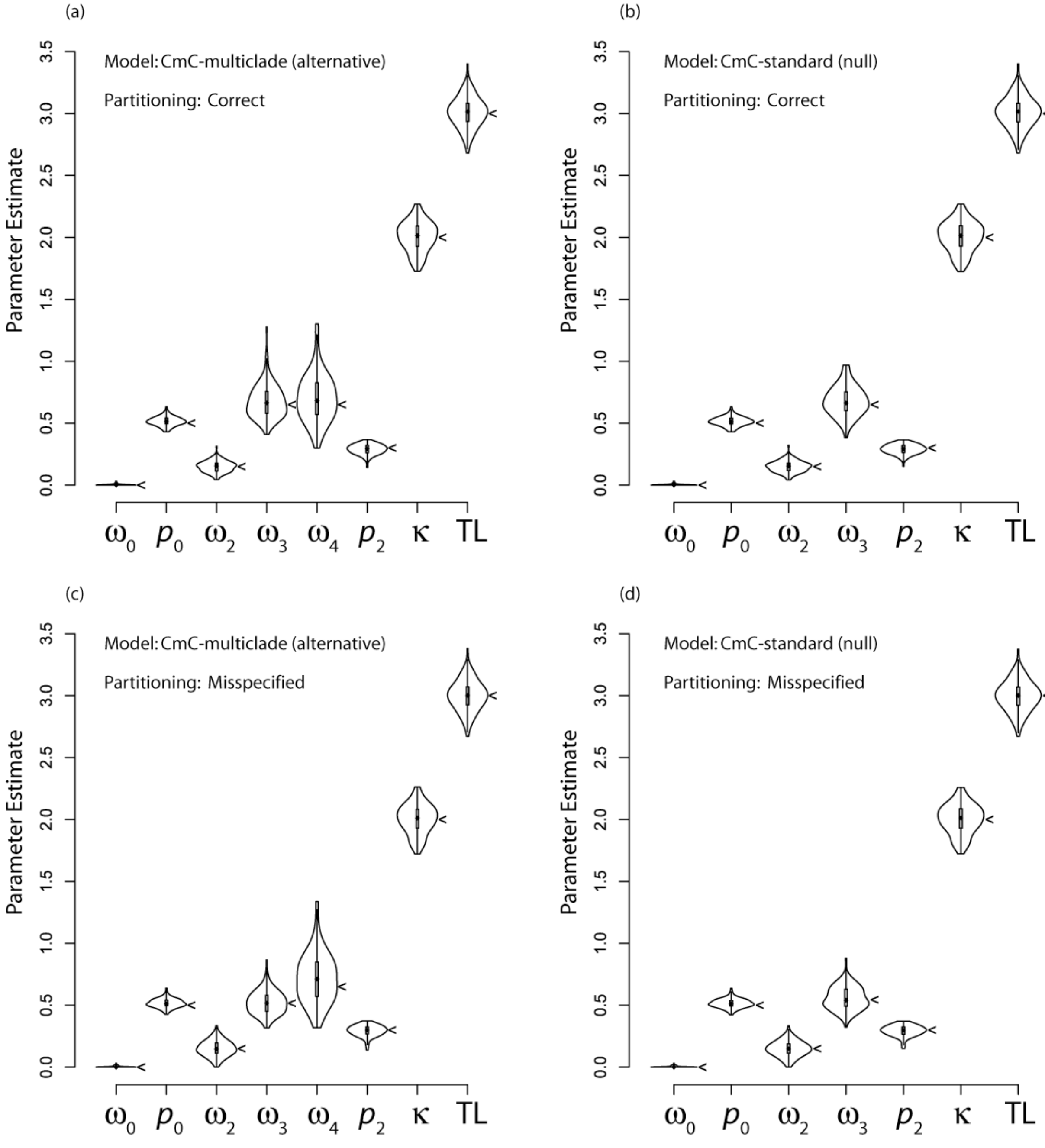

Supplementary Table 1—Inferred amino acid (AA) substitutions along the RH2aα and RH2aβ post-duplication branches. Node states were inferred using codeml given the estimated phylogeny (Fig. 1) and assuming the WAG+F+Γ amino acid substitution model. Support values (posterior probabilities) for ancestral node states are provided in parentheses.

Branch	Site	Substitution from…		Substitution to…
RH2aα Post-duplication branch	158	Leucine (0.996)	→	Phenylalanine (0.990)
	162	Isoleucine (0.945)	→	Valine (0.968)
	263	Isoleucine (0.782)	→	Valine (0.973)
	290	Isoleucine (0.811)	→	Leucine (0.989)
RH2aβ Post-duplication branch	27	Proline (0.865)	→	Threonine (0.989)
	31	Methionine (0.834)	→	Leucine (1.000)
	32	Valine (0.998)	→	Alanine (1.000)
	36	Isoleucine (0.686)	→	Phenylalanine (1.000)
	37	Tyrosine (0.845)	→	Phenylalanine (0.999)
	39	Isoleucine (0.388)	→	Leucine (1.000)

NOTE—Site numbering follows that of bovine RH1 opsin.

Supplementary Table 2—Parameter estimates, log-likelihood scores, and AIC weights obtained from Clade model D (CmD) analyses of the RH2a data set. Models are ranked according to AIC score. Unlike Clade model C (employed in the main text), CmD places no constraints on the ω parameter estimates for any of the site classes. LRT *P* values for various comparisons are included in Supplementary Table 3.

Model (n.p.)	SC 0		SC 1		SC 2		κ	*ln*L	Δ AIC	AIC weight
	ω_0	p_0	ω_1	p_1	ω_2, ω_3, ω_4	p_2				
CmD $\alpha\beta_{MVR}$ & β_T (40)	0.0000	0.6776	0.1835	0.1525	ω_2: 0.5166<br>ω_3: 1.2793<br>ω_4: 3.0207	0.1700	1.7049	-3773.5967	—	0.5676
CmD αβ (39)	0.0000	0.6924	0.2509	0.1676	ω_2: 0.5334<br>ω_3: 1.8185	0.1400	1.7024	-3775.2576	1.32	0.2931
CmD α & β (40)	0.0000	0.6891	0.2327	0.1623	ω_2: 0.5289<br>ω_3: 1.5740<br>ω_4: 1.8624	0.1487	1.7033	-3775.1872	3.18	0.1157
CmD β (39)	0.0000	0.4308	0.0308	0.3559	ω_2: 0.5500<br>ω_3: 1.5453	0.2133	1.7031	-3778.3642	7.54	0.0131
CmD β_T (39)	0.0000	0.6456	0.0981	0.1495	ω_2: 0.6066<br>ω_3: 2.6893	0.2050	1.6969	-3778.6277	8.06	0.0101
CmD α (39)	0.0049	0.7407	0.4699	0.2133	ω_2: 0.9245<br>ω_3: 3.8554	0.0460	1.6907	-3782.5678	15.94	0.0002
M2 (k = 2) (36)	0.0121	0.7821	0.6749	0.2179	-	-	1.6960	-3786.0205	16.85	0.0001
M3 (k = 3) (38)	0.0000	0.6888	0.2098	0.1458	ω_2: 0.7653	0.1654	1.6940	-3785.8744	20.56	0.0000

NOTE—n.p. = number of parameters; SC = site class.

Supplementary Table 3—Likelihood ratio test (LRT) *P* values for nested Clade model D (CmD) comparisons. Degrees of freedom for each LRT are indicated in parentheses.

Alternative \ Null	M3 (k = 2)	M3 (k = 3)	CmD α	CmD β	CmD αβ	CmD β_T
CmD $\alpha\beta_{MVR}$ & β_T	0.0001 (4)	< 0.0001 (2)	-	-	0.0684 (1)	0.0015 (1)
CmD α & β	0.0002 (4)	< 0.0001 (2)	0.0001 (1)	0.0117 (1)	0.7076 (1)	-
CmD αβ	0.0001 (3)	< 0.0001 (1)	-	-	-	-
CmD α	0.0750 (3)	0.0101 (1)	-	-	-	-
CmD β	0.0016 (3)	0.0001 (1)	-	-	-	-
CmD β_T	0.0020 (3)	0.0001 (1)	-	-	-	-

Supplementary Table 4—Phylogenetic location of inferred substitutions at the 27 sites listed in Table 5. These sites were identified by Bayes empirical Bayes analysis as members of the 'divergently evolving' site class under the CmC $\alpha\beta_{MVR}$ & β_T model with posterior probability (PP) > 0.75. Node states were inferred using codeml given the estimated phylogeny (Fig. 1) and assuming the WAG+F+Γ amino acid substitution model. The phylogenetic locations of these substitutions are shown graphically in Figure 4.

Branch: ancestral node..descendent node (see labeled Newick format tree, below)	Inferred substitutions Site: From… (PP) → To… (PP)
1: 19..20	165: L(0.854)→M(0.988)<br>218: L(0.886)→I(0.989)<br>284: T(0.881)→S(0.926)
2: 20..3 (Cfr_RH2A)	31: M(0.836)→L<br>36: I(0.685)→F<br>56: G(1.000)→A<br>99: V(0.541)→F<br>107: P(0.999)→A<br>109: F(0.999)→A<br>149: T(0.954)→S<br>179: I(0.997)→L<br>273: G(0.999)→A<br>282: S(0.981)→Y<br>304: V(0.996)→I<br>335: T(0.998)→A
3: 20..21	-
4: 21..22	27: P(0.865)→T(0.989)<br>31: M(0.834)→L(1.000)<br>36: I(0.686)→F(1.000)
5: 22..5 (Oni_RH2Ab_DQ235682)	22: S(1.000)→N<br>27: T(0.989)→S

	99: V(0.558)→I<br>107: P(0.996)→T<br>277: M(0.967)→L<br>282: S(0.998)→A
6: 22..23	-
7: 23..24	56: G(1.000)→S(0.997)<br>99: V(0.558)→F(0.999)
8: 24..8 (Tdu_RH2_AY775089)	158: L(0.999)→F<br>162: I(0.997)→V<br>304: V(0.996)→I
9: 24..25	149: T(0.967)→S(1.000)
10: 25..13 (Tin_RH2Ab_GQ422501)	-
11: 25..26	-
12: 26..10 (Ppu_RH2Ab_AY673699)	179: I(0.995)→L
13: 26..27	56: S(0.747)→G(1.000)<br>107: P(0.999)→S(1.000)
14: 27..1 (Mze_RH2Ab_DQ088650)	-
15: 27..11 (Mau_RH2Ab_AY775091)	218: I(1.000)→T
16: 23..28	107: P(0.996)→A(0.992)<br>109: F(1.000)→S(0.998)<br>112: I(1.000)→V(0.971)<br>165: M(1.000)→V(0.989)<br>214: V(1.000)→F(0.996)<br>335: T(1.000)→A(0.989)

17: 28..6 (Nbr_RH2_AY775068)	24: F(0.923)→Y<br>179: I(0.968)→L<br>263: I(0.928)→V
18: 28..7 (Ove_RH2_AY775067)	122: E(0.992)→Q<br>207: M(0.993)→L<br>213: F(0.997)→V<br>273: G(0.999)→V<br>277: M(0.958)→L<br>290: I(0.990)→L<br>304: V(0.972)→I
19: 21..29	158: L(0.996)→F(0.990)<br>162: I(0.945)→V(0.968)<br>263: I(0.782)→V(0.973)<br>290: I(0.811)→L(0.989)
20: 29..4 (Oni_RH2Aa_DQ235683)	22: S(0.987)→N<br>36: I(0.917)→T<br>277: M(0.948)→L<br>282: S(0.976)→A
21: 29..30	27: P(0.863)→T(1.000)<br>99: V(0.611)→L(1.000)<br>149: T(0.940)→S(1.000)<br>284: S(0.921)→T(1.000)
22: 30..9 (Ppu_RH2Aa_GQ422490)	-
23: 30..31	218: I(1.000)→V(1.000)
24: 31..2 (Mze_RH2Aa_DQ088651)	-
25: 31..12 (Mau_RH2Aa_GQ422483)	214: V(1.000)→I<br>263: V(1.000)→I

26: 31..14 (Tin_RH2Aa_GQ422498)	-
27: 19..32	214: V(0.727)→I(0.985)<br>282: S(0.913)→A(0.987)<br>290: I(0.756)→L(0.992)
28: 32..15 (Pre_RH2_1_DQ234859)	99: V(0.648)→L<br>158: L(0.994)→T<br>179: I(0.986)→L<br>263: I(0.746)→V<br>335: T(0.990)→A
29: 32..16 (Lgo_RH2_AY296739)	277: M(0.649)→L
30: 19..33	36: I(0.836)→M(0.996)<br>99: V(0.535)→F(0.998)<br>162: I(0.945)→V(0.989)<br>263: I(0.723)→V(0.991)<br>277: M(0.689)→L(0.995)
31: 33..17 (Ola_RH2B_AB223054)	149: T(0.632)→S
32: 33..18 (Ola_RH2C_AB223055)	112: I(0.962)→V<br>122: E(0.985)→Q<br>213: F(0.992)→C<br>214: V(0.815)→I<br>273: G(0.992)→A

NOTE—Site numbering follows that of bovine RH1 opsin. Node labeling follows: ((3_Cfr_RH2A, ((5_Oni_RH2Ab_DQ235682, ((8_Tdu_RH2_AY775089, (13_Tin_RH2Ab_GQ422501, (10_Ppu_RH2Ab_AY673699, (1_Mze_RH2Ab_DQ088650, 11_Mau_RH2Ab_AY775091) 27) 26) 25) 24 , (6_Nbr_RH2_AY775068, 7_Ove_RH2_AY775067) 28) 23) 22 , (4_Oni_RH2Aa_DQ235683, (9_Ppu_RH2Aa_GQ422490, (2_Mze_RH2Aa_DQ088651, 12_Mau_RH2Aa_GQ422483, 14_Tin_RH2Aa_GQ422498) 31) 30) 29) 21) 20 ,

(15_Pre_RH2_1_DQ234859, 16_Lgo_RH2_AY296739) 32 , (17_Ola_RH2B_AB223054, 18_Ola_RH2C_AB223055) 33) 19;
